# 3D endothelial network formation in hydrogels improved by stromal cells and specific growth factors

**DOI:** 10.1038/s41598-025-25381-x

**Published:** 2025-11-24

**Authors:** Ivana Acimovic, Vaclav Chochola, Jose Luis Herrera, Ales Hampl, Josef Jaros

**Affiliations:** 1 Department of Histology and Embryology, Faculty of Medicine, Masaryk University, Brno, 62500 Czech Republic; 2 Cell and Tissue Regeneration, International Clinical Research Center, St. Anne’s University Hospital, Brno, 60200 Czech Republic; 3 Division of Pulmonary, Critical Care and Sleep Medicine, University of Miami Miller School of Medicine, Miami, FL USA

**Keywords:** Vascularization, Co-culture, Hydrogel, Growth factor, Xeno-free, Biological techniques, Biotechnology, Cell biology

## Abstract

The limiting factor in the current state-of-the-art bioengineering of human tissues/organoids in vitro is the lack of functional vasculature that would support growth, metabolic waste removal, and oxygen supply of the formed constructs. The three-dimensional (3D) tissues can be formed by utilization of specific cell types, different hydrogels and scaffolds that provide a physical support, in addition to specific medium composition. Establishment of chemically defined xeno-free conditions is of great interest when keeping in mind the transition from basic research to therapeutic applications. In this study, we present the formation of vascular network of human umbilical vein endothelial cells co-cultured with stromal cells (SCs), human dental pulp SCs or human adipose SCs, in natural hydrogels (Matrigel, GelMA) as well as xeno-free VitroGel hydrogels. As medium composition plays a pivotal role in the successful generation of biological constructs in vitro, we evaluated the potency of specific growth factors (GFs) to induce vascular network formation in 3D hydrogels. We confirmed that vascular endothelial growth factor (VEGF), the most used pro-angiogenic factor, is not mandatory in culture medium for vascular network formation. Rather the endothelial cell (EC) network in co-culture of ECs and SCs can be formed through stimulation of SCs with GFs such as insulin-like growth factor 1 (IGF1), basic fibroblast growth factor (FGF2), and epidermal growth factor (EGF) regardless of serum presence in the medium. Also, we tested completely chemically defined medium that, when used together with xeno-free hydrogel, presents a step forward to application of such tissue constructs in translational regenerative medicine.

**Supplementary Information:**

The online version contains supplementary material available at 10.1038/s41598-025-25381-x.

## Introduction

In recent years, there has been a growing interest in the formation of vascular endothelial networks in three-dimensional (3D) in vitro models^1^. This is driven by the need for more complex models to study cell-cell interactions, mechanisms of vasculogenesis and angiogenesis, as well as the development of advanced strategies for vascularization of tissue constructs in tissue engineering. Current approaches for creating tissue constructs involve the culture and differentiation of stem cells in the form of organoids growing inside hydrogels or porous scaffolds. The most effective and widely used hydrogel for organoid production has been commercially available Matrigel^2,3^, which is derived from the basement-membrane matrix extracted from Engelbreth-Holm-Swarm (EHS) mouse sarcomas.

Traditionally, the tube formation assay, a key test for evaluating the effects of pro- and anti-angiogenic factors on the arrangement of endothelial cells (ECs), has been performed in 2D, on the surface of Matrigel^4^. However, 3D endothelial networks are commonly formed using other natural, fibrous hydrogels such as fibrin and collagen^5–10^, whereas little is known about the ability of Matrigel to serve as a sole 3D matrix for network formation^11^. In fact, endothelial cells in bulk Matrigel typically fail to form extensive or stable networks, even when seeded as spheroids, and pre-formed vessels collapse rapidly under flow conditions^12^. While stromal cell support of 3D endothelial networks has been demonstrated in fibrous or hybrid hydrogels, evidence for this in pure Matrigel remains scarce, and addressing this gap became one of the goals of our study.

In 3D endothelial assays more broadly, stromal cells (SCs) play an essential role by providing both paracrine and structural support^13^. During the process of angiogenesis in vivo, ECs recruit the SCs, which is considered a step of vessel maturation^14^. Human dental pulp-derived stromal cells (DPSCs) isolated from the third molars and adipose tissue-derived stromal cells (ASCs) isolated from lipoaspirates have been of huge interest in regenerative medicine research in recent years because of their easy isolation and multipotency. They can be differentiated into cells of different lineages (i.e., adipocytes, chondrocytes, odontoblasts, ECs)^15,16^. Besides the differentiation potential, they are known by their secretome that contains the pro-angiogenic factors^17–19^.

A major challenge for 3D co-culture systems and the study of interactions between different cell types in vitro is to establish an appropriate medium and microenvironment that support not only cell survival but also other cell functions. SCs are commonly propagated in mesenchymal stem cell (MSC) medium, which includes 10% fetal bovine serum (FBS), representing a variable and undefined component^20^. On the other hand, ECs are typically cultured in media supplemented with bovine pituitary extract or commercially available almost fully defined endothelial growth medium (EGM2), which contains only 2% fetal calf serum (FCS)^21^. The easiest way for co-culture of ECs and SCs is to use one of these media or to mix them in different ratios^22^. However, it does not allow us to conclude how the individual components of the media influence each of the cell types due to complex cellular interactions at both physical and molecular levels.

In this study, we focused on investigating the effect of selected growth factors (GFs) and other components present in EGM2 on endothelial network formation using human umbilical vein endothelial cells (HUVECs) co-cultured with DPSCs or ASCs. Our goal was to identify the key drivers of angiogenesis, which could be used for enrichment of other culture media. We examined each of the GFs (insulin-like growth factor 1 (IGF1), epidermal growth factor (EGF), basic fibroblast growth factor (FGF2), vascular endothelial growth factor (VEGF)) from EGM2 medium individually and in combination with each other in order to see their effect on EC and SC co-cultures. The main goal was to define the minimal medium composition needed for vascular network formation in co-cultures that also might help as a starting point for development of medium composition for tri-cultures (i.e., ECs, SCs, and human pluripotent stem cell-derived organoids). Primarily, we focused on vascularization of Matrigel as the hydrogel that is the most widely used for embedding of organoids.

We established a co-culture system in which the evaluated hydrogel sample had always the same initial size, as opposed to Matrigel domes. The cylindrical shape of hydrogel with defined diameter, along with its placement in surrounding agarose, ensured a consistent supply of cell medium. This system offered several advantages, namely, standardized initial hydrogel dimensions and cell distribution in all experiments, enabling 3D cell organization, and comparative evaluation of shrinking of hydrogels caused by cell activity and evaluation of endothelial network parameters, particularly the length of protrusions.

We investigated three types of scaffolds with distinct properties – hydrogel, Matrigel, a solubilized basement membrane matrix from EHS mouse sarcoma, often used for stem cells’ culture and their differentiation; a porous scaffold, methacrylated gelatin (GelMA) with high stiffness to prevent gel remodeling by cells; and xeno-free hydrogels, VitroGel, enriched with several synthetic adhesion peptides. We demonstrated that endothelial network formation depends on the presence of specific medium components and the type of used hydrogel. Our findings represent a significant step towards production of vascularized constructs with potential clinical applications under xeno-free and chemically defined conditions.

## Materials and methods

### Cell culture

Human umbilical vein endothelial cells (HUVECs; Lonza, C2519A) were cultured at 37 °C in 5% CO_2_ on gelatin-coated cell culture dishes in Endothelial Cell Growth Medium 2 (EGM2) consisting of Endothelial Cell Basal Medium 2 (EBM; PromoCell, C-22211) and Endothelial Cell Growth Medium 2 Supplement Pack (PromoCell, C-39211). The final concentration of each component was 2% FCS, 5 ng/ml EGF, 10 ng/ml FGF2, 20 ng/ml IGF1, 0.5 ng/ml VEGF, 1 µg/ml ascorbic acid, 22.5 µg/ml heparin, 0.2 µg/ml hydrocortisone, and 1% Penicillin-Streptomycin (P/S; Biosera, XC-A4122). HUVECs were expanded and used for experiments up to passage 7.

VEGF was tested at concentrations of 0.5, 10, 50, and 100 ng/ml, where 0.5 ng/ml corresponds to the concentration present in EGM2 medium, 10 ng/ml reflects a commonly used dose in angiogenesis assays, and 50–100 ng/ml represent higher concentrations reported in 3D vascularization studies to enhance endothelial network formation^23,24^.

Isolation of adipose-derived stromal cells (ASCs) was performed in compliance with the ethical standards provided in the 1964 Declaration of Helsinki and its later amendments or comparable ethical standards and approved by the Ethics Committee St. Anne’s University Hospital Brno (8 V/2020). Written informed consent approving experimental use of extracted adipose tissue was obtained from the healthy donor before the liposuction procedure was applied. ASCs were isolated by centrifugation method from fat tissue described in^25^ and cryopreserved in passage 1.

Dental pulp-derived stromal cells (DPSCs; Lonza) and ASCs were expanded in Mesenchymal Stem Cell (MSC) medium composed of DMEM/F-12 (Gibco, 21331-020) supplemented with 10% FBS (Biosera, FB-1101), 1× GlutaMAX-1 (Gibco, 35050-061), and 1% P/S. Cells were used up to passage 12 for different experiments. For all cells, cell medium was changed every 2–3 days.

### Formation of HUVEC spheroids

Agarose microwells were casted in silicone molds (3D Petri Dishes, Microtissues) according to the manufacturer’s instructions. 256-microwell molds made of 2% (w/v) agarose (ITW Reagents, A8963) dissolved in 1× PBS were seeded with HUVECs to a final ratio of 200 cells/spheroid. After 24 h, the formed spheroids were collected by gentle pipetting.

### Preparation of 3D hydrogels and embedding of cells

To make the cylinder mold, wells of 24 well plate (24WP) were filled with 350 µl of 2% (w/v) agarose dissolved in 1× PBS. After the agarose had solidified, a 6 mm biopsy punch (Integra Miltex, 33–36) was used to make a hole in the central part of the well. The inner piece of agarose was removed while the agarose ring stayed. The agarose ring was then equilibrated in basal medium for at least 24 h.

Within the agarose rings, a 15 µl of Matrigel was solidified at the bottom to form a separating layer and 30 µl of Matrigel with embedded cells was gently added. The plate was incubated at 37 °C for 20 min, after which 1 ml of medium was added to each well.

HUVECs and DPSCs/ASCs were resuspended in growth factor reduced (GFR) Matrigel (Corning, 354230, protein 8.8 mg/ml) at 4 °C in several variations. HUVECs were embedded as single cells at a concentration 0.5 × 10^6^ or 5 × 10^6^ cells/ml, or as spheroids at a concentration of 0.5 × 10^6^ cells/ml (200 HUVECs per spheroid). DPSCs/ASCs were prepared at concentrations of 0.5, 1, 2 or 4.5 × 10^6^ cells/ml, or mixed either with HUVEC spheroids or single cells.

For co-culture setups with spatially separated HUVECs and DPSCs, Transwell inserts (Corning, Transwell Costar, Clear inserts, 0.4 μm pore size, 3470) were used. Inserts seeded with DPSCs at 4.5 × 10^6^ cells/insert were placed above the agarose ring and hydrogel with embedded HUVECs.

Gelatin methacryloyl (GelMA 170–195 Bloom, 60% DS, Sigma, 900741) was reconstituted in phenol red free DMEM (Gibco, 21041025) to 10% (w/v) solution at 37 °C and filtered through warm 0.22 μm syringe filter, protected from light. Poly(ethylene oxide) (PEO, Sigma, 182001, average Mv 300,000) was dissolved in phenol red free DMEM to 1.6% (w/v) solution. Next, lithium phenyl-2,4,6-trimethylbenzoylphosphinate photoinitiator (LAP, Sigma, 900889) was dissolved in PEO solution to the final concentration of 0.2% LAP with 1.6% PEO. Then, the solution was filtered through a 0.22 μm filter. Both stock solutions (10% GelMA and 0.2% LAP + 1.6% PEO) were kept at 4 °C protected from light and heated to 37 °C prior use. To produce final porous GelMA solution, stock solutions were freshly mixed in a 1:1 ratio^26^. HUVEC spheroids (0.5 × 10^6^ cells/ml, 200 HUVECs/spheroid) together with 4.5 × 10^6^ DPSCs/ml were resuspended in porous GelMA solution (GelMA + LAP + PEO). 15 µl of non-porous GelMA (GelMA + LAP) was added in the cylinder hole in 2% agarose and photo-crosslinked for 2 min at 400 nm light by UV lamp to form the bottom layer. 30 µl of porous GelMA with cells was pipetted on the top of bottom layer and photo-crosslinked by 400 nm light for 2 min. After that, 1 ml of medium was added per well.

VitroGel xeno-free hydrogels (TheWell Bioscience; VitroGel Angiogenesis Assay HC Kit (TWG011), VitroGel 3D High Concentration (TWG001), VitroGel COL High Concentration (TWG009), VitroGel RGD High Concentration (TWG003), VitroGel IKVAV High Concentration (TWG007), VitroGel YIGSR High Concentration (TWG008), VitroGel ORGANOID (VHM04-3)) were prepared according to the manufacture’s protocols. Briefly, the high concentration VitroGels (3D, COL, RGD, IKVAV, and YIGSR) were diluted with VitroGel Dilution Solution in a 1:2 ratio and mixed with the cell medium with cells (12 µl VitroGel hydrogel + 24 µl Dilution solution + 9 µl EBM with cells for the final volume of 45 µl loaded to the agarose cylinder mold). Angiogenesis Assay VitroGel was mixed in a 2:1 ratio with cells resuspended in supplement 1 (30 µl gel + 15 µl supplement 1 with cells) or diluted 1:1 with dilution solution and then mixed with the cells resuspended in supplement 1 (15 µl gel + 15 µl dilution solution + 15 µl supplement 1 with cells). For ORGANOID, the gel was mixed with cells resuspended in EBM in a 2:1 ratio. The gels were kept for 20 min at room temperature (RT) before the addition of cell culture media.

All hydrogel constructs were cultured at 37 °C for 7 days with medium changes every 2 days. Detailed composition of all used media is given in Tables S1-S3.

### Real-time PCR analysis

The samples HUVEC spheroids (0.5 × 10^6^ cells/ml; 200 HUVECs per spheroid) in co-culture with DPSCs (4.5 × 10^6^ cells/ml) in Matrigel in EGM2 were collected at days 1 and 7 for real-time PCR. The Matrigel with the embedded cells was removed from the agarose ring, collected in 15 ml tube with ice-cold 1× PBS, centrifuged at 400 g, 4 min, at 4 °C. This step was repeated 3 times. After removing the PBS, samples were lysed in RNA blue (Top-Bio). Total RNA was isolated according to the manufacturer’s instructions. 1 µg of total RNA was used for reverse transcription with High Capacity cDNA Reverse Transcription Kit (ThermoFisher Scientific, 4368814). Real-time PCR was performed using RT^2^ SYBR Green qPCR Mastermix (Qiagen, 330502) and specific forward and reverse primers in the final concentration of 0.5 µM. The list of used primers is given in the Table S4. Reactions were run at 95 °C for 10 min, followed by 45 cycles at 95 °C for 10 s, 60 °C for 10 s, and 72 °C for 10 s on LightCycler 480 II (Roche). Expression levels of *CXCL12*, *VWF*, *VEGFA*, *MKI67*, and *MMP2* were normalized to the housekeeping gene *GAPDH*. Relative gene expression was calculated by the 2^−ΔCt^ method. The experiments were done in triplicate.

### Fluorescent staining, confocal microscopy, and analysis of vascular network

Cells were washed with 1× PBS, fixed with 4% paraformaldehyde (PFA) in 1× PBS for 2 h at RT, and agarose rings were removed. Hydrogels were washed three times with 1× PBS. The samples were permeabilized with 0.1% Triton X-100 (Sigma, X-100) in 1× PBS for 5 min at RT, washed with 1× PBS for 15 min, and blocked with 5% BSA (Sigma, A9647) and 0.05% TWEEN 20 (Sigma, P1379) in 1× PBS for 1 h at RT on shaker. HUVECs were stained with Agglutinin from *Ulex europaeus*, Fluorescein (UEA, Vector Laboratories, FL-1061) at a final concentration of 10 µg/ml. Actin filaments and nuclei of all cells were counterstained with rhodamine phalloidin (Life Technologies, R415; 1:400) and 4′,6-diamidino-2-phenylindole (DAPI; 1 µg/ml), respectively, for 4 h at RT in gentle agitation. Samples were extensively washed with 0.05% Tween in 1× PBS at RT overnight. 1× PBS was changed before microscope observation.

To confirm the identity of ECs and SCs, the samples were immunostained with the primary antibodies CD31 (DAKO, M0823; 1:100) and α-SMA (Cell Signaling, 19245T; 1:100) in 5% BSA in 1× PBS at RT in gentle agitation overnight. Then, cells were washed extensively with 0.05% Tween in 1× PBS on the shaker at RT, followed by incubation with the secondary antibodies conjugated with Alexa Flour 488 (Invitrogen, A11001) and Alexa Fluor 568 (Invitrogen, A11036). Nuclei were counterstained with DAPI (1 µg/ml) in 0.05% Tween in 1× PBS shaking overnight at RT. Then, the samples were washed three times in 0.05% Tween in 1× PBS during 24 h.

Samples were imaged using a confocal microscope (Zeiss LSM 700). 11 z-planes (z scaling 5.158 μm) were acquired at different positions for each sample. Maximum Intensity Projection images were generated using Zen Black software (Carl Zeiss). Length of outgrowing protrusions of HUVECs in hydrogels was labeled as vascular network length and assessed with Angiogenesis Analyzer plugin in ImageJ^27^. Other parameters of the vascular network, such as the number of junctions, the number of meshes, and the number of branches, were also analyzed. The results were graphically represented using GraphPad Prism or the ggplot2 graphics package in R.

### Evaluation of hydrogel shrinkage

Bright field images of hydrogel cylinders were acquired for 7 consecutive days using Leica M165FC stereomicroscope equipped with a Leica DFC450 C digital camera. Images were taken at 2 different z positions. One image was focused on the outer border of hydrogel, while the other on the central part of hydrogel cylinder. Acquired images were analyzed to measure the area of the hydrogel, with the average of two measurements used for further evaluation. The hydrogel’s outline was manually traced using freehand selection tool in ImageJ to calculate its area. The final area of the hydrogel cylinders was expressed as a percentage of initial area.

### Statistical analysis

Data were collected from 2 to 16 replicates. Normality of data was evaluated with Kolmogorov-Smirnov test, using Prism (version 5.0; GraphPad). When the data passed the normality test, unpaired t-test was used to compare two groups while one-way ANOVA with Bonferroni’s multiple comparison *post hoc* test was used in the case of multiple groups. If the data did not have a normal distribution, the Mann-Whitney test was used to compare two groups or Kruskal-Wallis with Dunn’s multiple comparison test in the case of multiple groups. Data are shown as mean ± standard deviation (SD) or as Tukey-style boxplots with median, box representing the interquartile range (IQR), the whiskers extending to 1.5× IQR, and points outside this range representing outliers, unless otherwise stated in the figure legend. * *p* < 0.05, ** < 0.01, *** *p* < 0.001, **** *p* < 0.0001.

## Results

### Matrigel remodeling by DPSCs enables HUVEC network formation in co-cultures

**Fig. 1:**
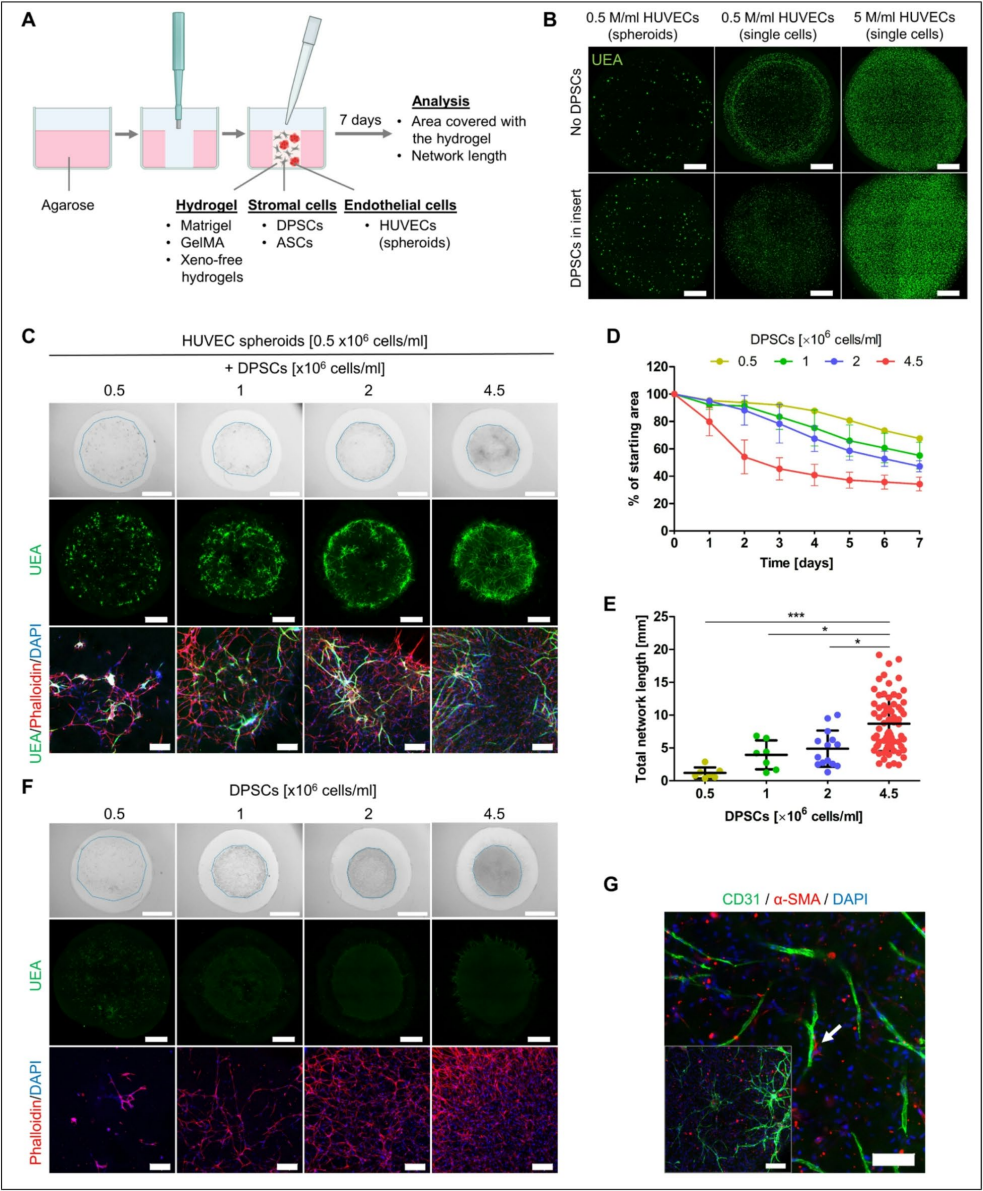
Co-cultures of HUVECs and DPSCs in 3D Matrigel in EGM2 medium. (**A**) Schematics of experimental setup (created with BioRender.com). (**B**) Maximum intensity projections of confocal z-stack images showing HUVECs cultured within Matrigel for 7 days as spheroids or single cells at concentrations 0.5 × 10^6^ cells/ml and 5 × 10^6^ cells/ml without DPSCs (upper) or with 4.5 × 10^6^ DPSCs/ml placed in inserts (lower). Endothelial marker Ulex Europaeus Agglutinin I (UEA, green). Scale bar is 1 mm. (**C**) Co-culture of HUVEC spheroids with 0.5, 1, 2, and 4.5 × 10^6^ DPSCs/ml within 3D Matrigel for 7 days. Bright field images (upper), maximum intensity projections of confocal z-stack images showing the whole hydrogel (middle), maximum intensity projections of z-stack confocal images showing a detail (lower). Endothelial marker UEA (green), actin (phalloidin rhodamine, red), nuclei (DAPI, blue). Scale bars of 2 mm (upper), 1 mm (middle), 200 μm (lower). (**D**) Area covered with 3D Matrigel cylinder during 7 days of culture shown as percentage of initial area. HUVEC spheroids were in co-culture with different numbers of DPSCs: 0.5 × 10^6^ (*n* = 2), 1 × 10^6^ (*n* = 2), 2 × 10^6^ (*n* = 3), and 4.5 × 10^6^ cells/ml (*n* = 16). Data are shown as mean ± SD. (**E**) Total length of vascular network when HUVEC spheroids were seeded with 0.5 × 10^6^ (*n* = 7), 1 × 10^6^ (*n* = 7), 2 × 10^6^ (*n* = 14), and 4.5 × 10^6^ cells/ml (*n* = 69). Data are shown as mean ± SD. Kruskal-Wallis test with Dunn’s multiple comparison test was used for statistical analysis. * *p* < 0.05, *** *p* < 0.001. (**F**) DPSCs grown within 3D Matrigel. Bright field images (upper), maximum intensity projections of tiled confocal z-stack images (middle), maximum intensity projections of z-stack confocal images (lower). Scale bar 2 mm (upper), 1 mm (middle), 200 μm (lower). (**G**) Immunostaining of co-cultured HUVEC spheroids with 4.5 × 10^6^ DPSCs/ml in 3D Matrigel for CD31 (green), alpha-smooth muscle actin (α-SMA, red), and nuclei (DAPI, blue). Scale bar is 200 μm on the image in the corner and 100 μm on the image showing a detail. The white arrow pointing to the interaction of DPSC with HUVEC network.

Matrigel is broadly used for culture of various cell types, including stem cells. ECs are typically subjected to two-dimensional (2D) tube assay to showcase their ability to form vascular networks under planar culture conditions. To test Matrigel as an appropriate microenvironment for 3D vascular network formation, we embedded HUVECs into cylindrical Matrigel using a 6 mm diameter agarose mold (Fig. 1A) and cultured them in EGM2 medium for 7 days.

Initially, we seeded HUVECs at concentrations 0.5 × 10^6^ and 5 × 10^6^ cells/ml, both, as single cells and as spheroids (200 cells/spheroid). However, HUVECs failed to form vascular networks under these conditions (Fig. 1B). Since DPSCs are known to secrete pro-angiogenic factors^17,18^, we investigated whether DPSC-conditioned medium might support network formation in 3D Matrigel. We seeded DPSCs into an insert placed in the same well above the Matrigel cylinder containing HUVECs. After seven days, HUVECs failed to form networks in this setup as well, whether seeded as spheroids or single cells (Fig. 1B). Instead, HUVECs remained rounded, failing to migrate, connect, or form networks. The size and shape of Matrigel cylinder also remained unchanged.

Therefore, we further examined how the physical presence of DPSCs in the hydrogel would impact the behavior of HUVECs. In planar conditions, we observed that DPSCs joined the HUVEC network formed on the Matrigel layer (Fig. S1). This is consistent with previous studies showing that DPSCs act as a network stabilizer^28^. In 3D Matrigel, we tested different concentrations of DPSCs seeded in hydrogel together with HUVEC spheroids (Fig. 1C). Spheroids were employed to facilitate cell-cell interactions, especially during the initial days of cell culture. An increasing concentration of DPSCs within the hydrogel (0.5, 1, 2 and 4.5 × 10^6^ cells/ml) led to more extensive remodeling of Matrigel, which was assessed by measuring changes in the hydrogel’s total area over time (Fig. 1D). When 0.5 × 10^6^ DPSCs/ml were used, the area covered with the hydrogel gradually decreased after 7 days to 67.52 ± 0.73% of the initial area. The most pronounced remodeling occurred at the highest DPSC concentration (4.5 × 10^6^ DPSCs/ml), resulting in the hydrogel covering 34.28 ± 4.91% of the original area. DPSCs exhibited a rapid reduction in hydrogel area especially during the first three days of culture. On day 7, we measured the length of the formed vascular network, a key parameter indicating the efficiency of vascularization (Fig. 1E). A significantly greater network length of 8.70 ± 4.19 mm was observed when 4.5 × 10^6^ DPSCs/ml were used, in contrast to 4.88 ± 2.76, 3.95 ± 2.20, and 1.19 ± 0.84 mm achieved when 2, 1, and 0.5 × 10^6^ DPSCs/ml were used in the hydrogel, respectively. Furthermore, we confirmed our observations when DPSC were co-cultured with HUVECs seeded as single cells (0.5 × 10^6^ HUVECs/ml) (Fig. S2A). The trend of decreasing hydrogel area and increasing total vascular network length with higher DPSC numbers resembled that observed when HUVEC spheroids were used (Fig. S2B, C). The same trend of area reduction was observed when DPSCs were seeded alone in Matrigel (Fig. 1F), indicating their hydrogel remodeling ability. Importantly, with DPSCs being the single cell type within the gel we could verify the specificity of UEA staining (Fig. 1F).

In the co-culture, some DPSCs that were in contact with HUVECs expressed the alpha-smooth muscle actin (α-SMA), suggesting a maturation process (Fig. 1G).

To gain mechanistic insight into how stromal–endothelial co-cultures remodel Matrigel, we performed qPCR analysis of constructs containing HUVECs and DPSCs at day 1 and day 7 of culture. By day 7, we observed increased expression of *CXCL12*, a stromal cell–derived factor known to stimulate endothelial proliferation and angiogenesis, along with higher levels of *VEGFA* and the endothelial marker *VWF*. Expression of *MKI67* decreased over time, indicating reduced proliferation as cultures stabilized, whereas MMP2 expression increased, consistent with active matrix remodeling. Together, these changes support a model in which stromal–endothelial interactions promote vascular network formation through coordinated paracrine signaling and extracellular matrix (ECM) modification (Fig. S3).

These results highlight the necessity of SCs in Matrigel to establish network support for HUVECs and promote matrix remodeling. The degree of remodeling and vascularization positively correlates with DPSC concentration.

### Medium composition influences the DPSCs distribution within the hydrogel and HUVEC network formation

**Fig. 2:**
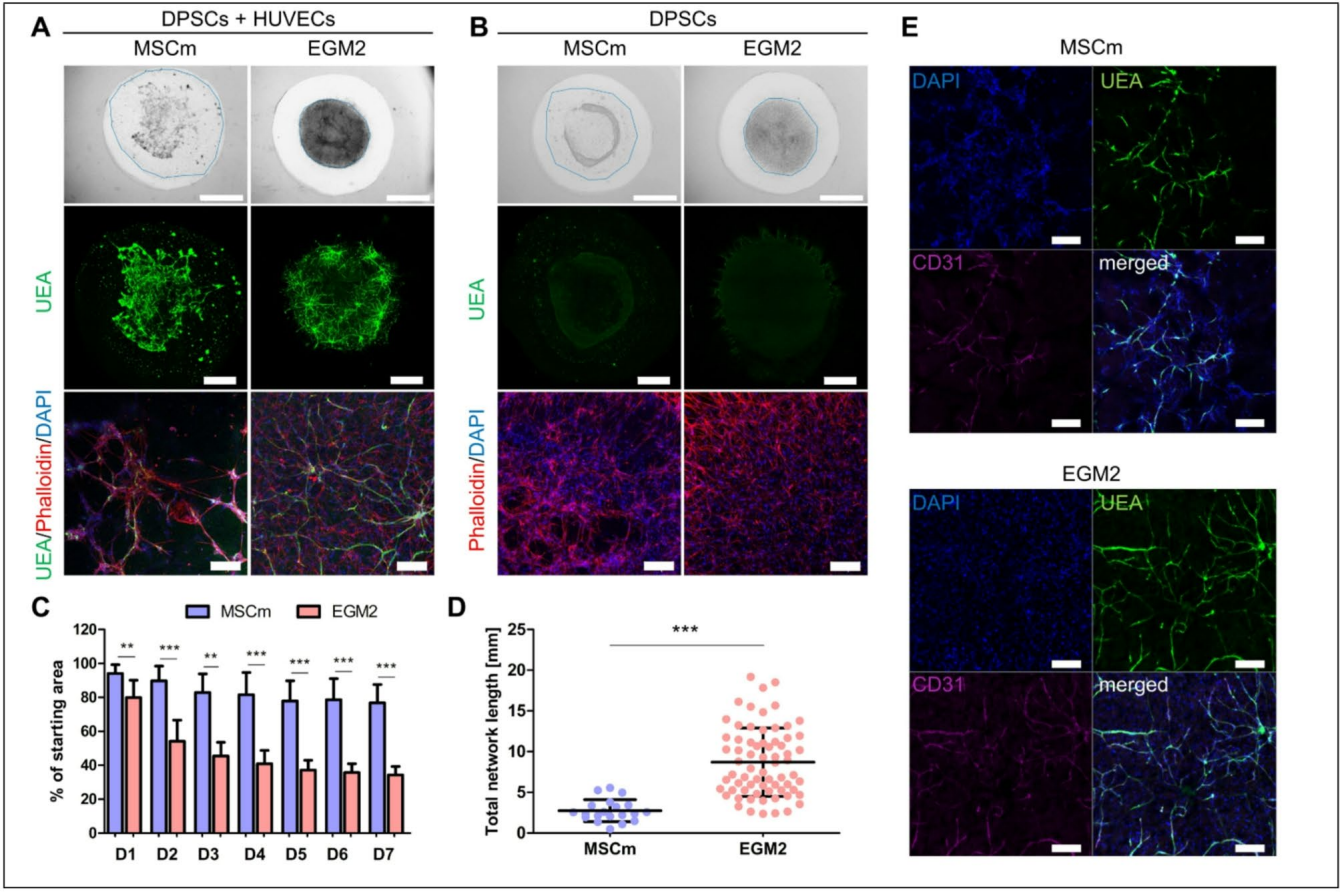
HUVECs and DPSCs co-cultured within 3D Matrigel in MSC or EGM2 medium. (**A**) 0.5 M/ml HUVECs in the form of spheroids with 4.5 M/ml DPSCs in MSC medium (MSCm, left) or EGM2 medium (right) at day 7 of cultivation. Bright field images of Matrigel with embedded cells (upper), maximum intensity projections of tiled confocal z-stack images (middle), maximum intensity projections of z-stack confocal images (lower). Staining with endothelial marker Ulex Europaeus Agglutinin I (UEA, green), actin with phalloidin rhodamine (red) and nuclei with DAPI (blue). Scale bar is 2 mm (upper), 1 mm (middle), 200 μm (lower). (**B**) 4.5 M/ml DPSCs in MSC medium (left) or EGM2 medium (right) at day 7 of cultivation. Bright field images of 3D Matrigel (upper), maximum intensity projections of tiled confocal z-stack images of 3D Matrigel (middle), maximum intensity projections of z-stack confocal images (lower). Staining with UEA (green), actin with phalloidin rhodamine (red) and nuclei with DAPI (blue). Scale bar is 2 mm (upper), 1 mm (middle), 200 μm (lower). (**C**) Area covered with 3D Matrigel during the course of 7 days in MSC (*n* = 5) and EGM2 medium (*n* = 16). Data are shown as mean ± SD. Unpaired t test (D1, D2, D4, D5, D6 and D7) or Mann Whitney test (D3) were used for statistical analysis. ** *p* < 0.01, *** *p* < 0.001. (**D**) Total network length formed in MSC medium (*n* = 20) and EGM2 medium (*n* = 69). Data are shown as mean ± SD. Mann Whitney test was used for statistical analysis. *** *p* < 0.001. (**E**) Maximum intensity projections of z-stack confocal images of 3D Matrigel with co-cultures of HUVEC spheroids and DPSCs in MSC medium (upper) or EGM2 medium (lower) stained with UEA (green) and CD31 (magenta). Nuclei stained with DAPI (blue). Scale bar is 200 μm.

We showed that HUVECs need a presence of DPSCs to form a network in Matrigel in EGM2 medium. We also asked to what extent the success of network formation is affected by the composition of the medium. Thus, we used the optimal condition from our previous experiment (HUVEC spheroids, 0.5 × 10^6^ cells/ml in co-culture with 4.5 × 10^6^ DPSCs/ml as single cells) to compare EGM2 medium and MSC medium. The distribution of cells in the hydrogel and the overall hydrogel remodeling differed significantly between the two tested media (Fig. 2A–D). While DPSCs in EGM2 medium were distributed throughout the hydrogel, DPSCs cultured in MSC medium formed the bundle-like structures that were centrally located within the hydrogel (Fig. 2A,B). There was also a significant difference in the area covered with Matrigel during 7 days (Fig. 2C). In MSC medium, the area covered with hydrogel changed by only 20% from baseline values compared with the extensive remodeling and reduction in hydrogel area in the EGM2. Subsequently, the spreading and distribution of DPSC influenced HUVEC network formation and there was a significant difference in the total length of the network formed in EGM2 compared to MSC medium (Fig. 2D).

Since DPSCs are known for their capacity to differentiate into specific cell lineages depending on the medium composition, we wanted to determine if DPSCs also contributed to the formed vascular networks by differentiating into ECs. DPSCs seeded alone in Matrigel did not express the endothelial marker UEA in MSC or EGM2 medium (Figs. 1F and 2B). Furthermore, in co-cultures with HUVECs, DPSCs did not express another endothelial marker, CD31, that specifically stained HUVEC network (Fig. 2E).

### Medium supplements FCS, ascorbic acid, heparin, and hydrocortisone have insignificant effect on HUVEC network formation

The formation of HUVEC networks in co-culture with DPSCs was more effectively promoted in EGM2 compared to MSC medium. To determine which specific components of EGM2 are the main drivers of this effect, we first focused on analyzing the role of medium supplements fetal calf serum (FCS), heparin (Hep), ascorbic acid (AA), and hydrocortisone (HC). To isolate the effect of their withdrawal in a minimal medium, we utilized endothelial basal medium (EBM), which included all 4 supplements but lacked the growth factors (EBM + FCS + Hep + AA + HC, further labelled only as EGM2-GFs). When we maintained co-cultures in EBM supplemented with different combinations of three out of four supplements (Hep + AA + HC (-FCS), FCS + Hep + HC (-AA), FCS + AA + HC (-Hep), FCS + Hep + AA (-HC)), we observed that none of these combinations supported the formation of HUVEC networks. Albeit the supplements are important for culturing and expansion of ECs, for which the EGM2 is optimized, this suggests that for the network formation, the role of the individual GFs might play a bigger role. In contrast to the spreading of DPSCs observed in EGM2, they did not disperse extensively throughout the hydrogel in the depleted supplement combinations (Fig. S4). Additionally, the area covered with Matrigel remained close to the initial size (Fig. S4B). Slight remodeling of hydrogel was observed in the absence of FCS or HC in the medium, resulting in 72.27 ± 8.12% and 76.19 ± 13.64% coverage, respectively. Under these two conditions, DPSCs managed to spread to a greater extent throughout the hydrogel compared to the conditions where FCS and HC were present together (EGM2-GFs, -AA, and -Hep) which also supported occasionally longer HUVEC sprouts from the spheroids (Fig. S4A). However, there were no differences in total HUVEC network length between the tested conditions that all were significantly lower than the total network length in EGM2 (Fig. S4C).

**Fig. 3:**
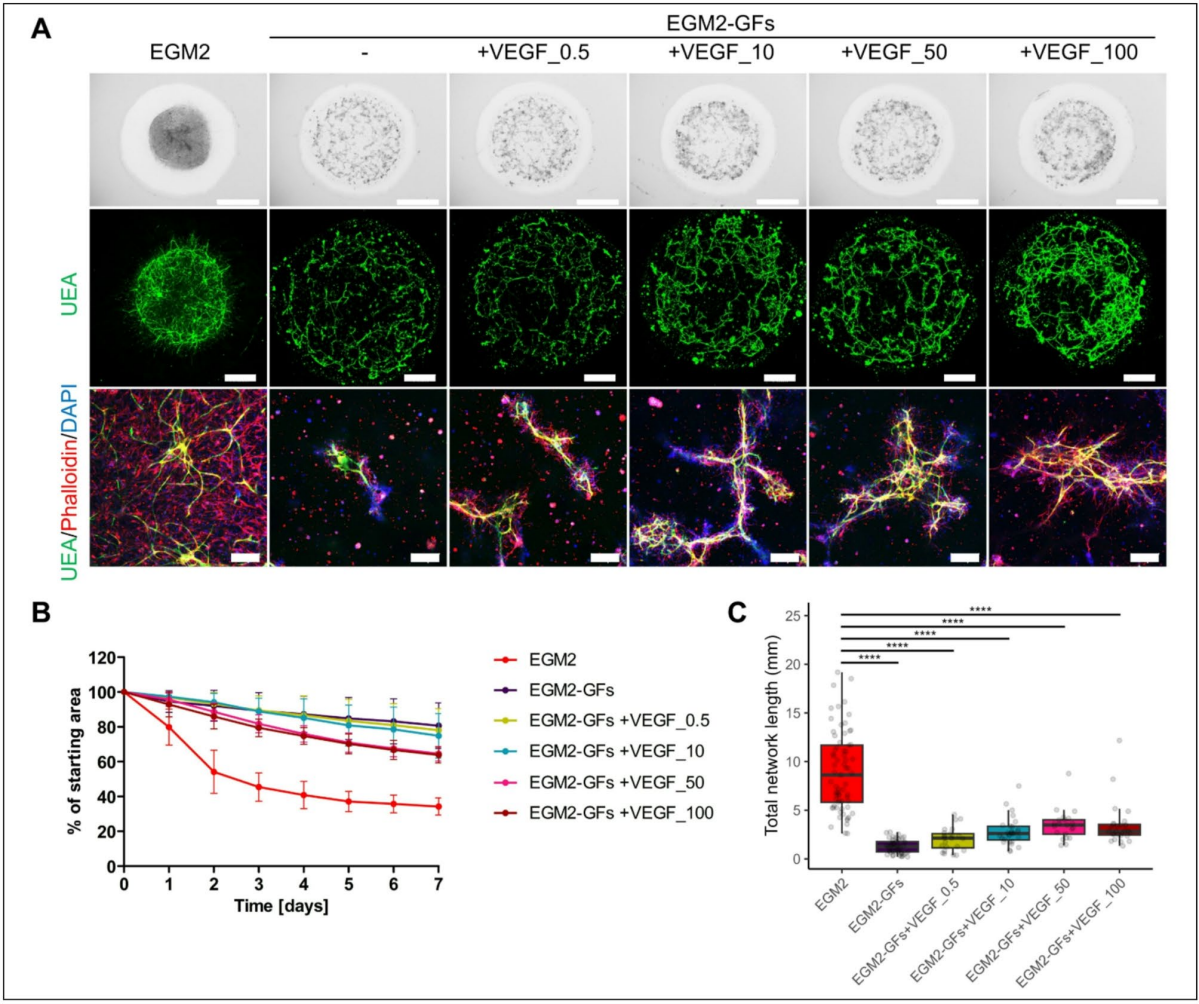
Effect of growth factor VEGF on HUVEC network formation in co-cultures with DPSCs in 3D Matrigel. (**A**) HUVEC spheroids in co-culture with DPSCs at day 7 of cultivation in media: EGM2, EGM2 without growth factors (EGM2-GFs), EGM2-GFs with 0.5, 10, 50 and 100 ng/ml VEGF (VEGF_0.5, VEGF_10, VEGF_50 and VEGF_100, respectively). Bright field images of 3D Matrigel (upper), maximum intensity projections of tiled confocal z-stack images of 3D Matrigel (middle), maximum intensity projections of z-stack confocal images (lower). Staining for endothelial marker Ulex Europaeus Agglutinin I (UEA, green), actin stained with phalloidin rhodamine (red) and nuclei with DAPI (blue). Scale bar is 2 mm (upper), 1 mm (middle), 200 μm (lower). (**B**) Area covered with 3D Matrigel during the course of 7 days in EGM2 (*n* = 16), EGM2-GFs (*n* = 6), VEGF_0.5 (*n* = 4), VEGF_10 (*n* = 4), VEGF_50 (*n* = 3) and VEGF_100 (*n* = 3). Data are shown as mean ± SD. (**C**) Total length of formed HUVEC network in EGM2 (*n* = 69), EGM2-GFs (*n* = 48), VEGF_0.5 (*n* = 24), VEGF_10 (*n* = 28), VEGF_50 (*n* = 21) and VEGF_100 (*n* = 27). Data are shown as Tukey-style boxplots with the median, IQR, whiskers (1.5 × IQR) and outliers. Each of the tested media was compared to EGM2 and Mann Whitney test was used for statistical analysis. **** *p* < 0.0001.

### Growth factors IGF1 and FGF2 are the main inducers of HUVEC network formation in co-cultures with DPSCs in 3D Matrigel

**Fig. 4:**
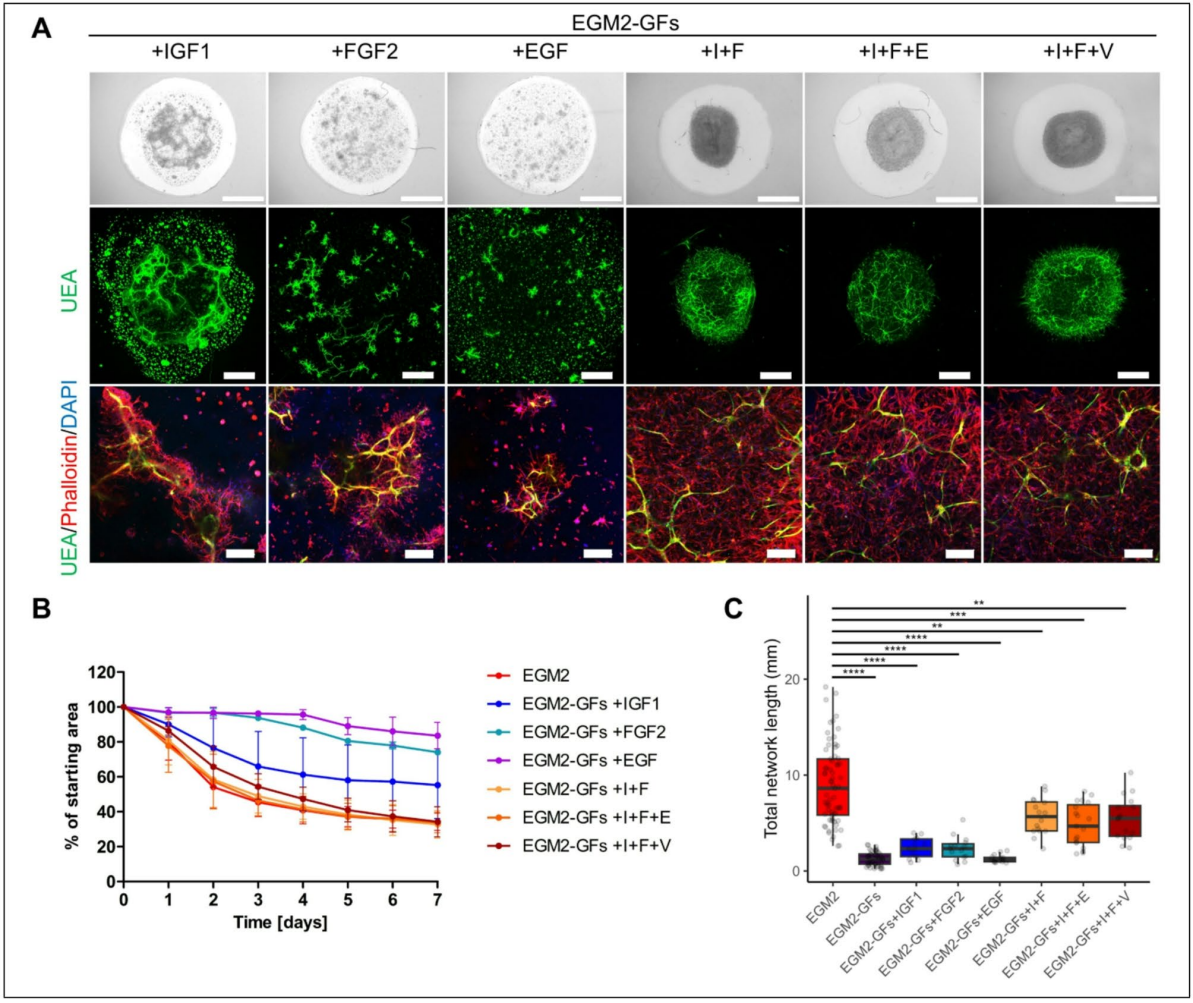
Effect of growth factors IGF1, FGF2 and EGF on HUVEC network formation in co-cultures with DPSCs in 3D Matrigel. (**A**) HUVEC spheroids in co-culture with DPSCs at day 7 of cultivation in media: EGM2-GFs supplemented with IGF1, FGF2, EGF, together IGF1 and FGF2 (I + F), together IGF1, FGF2 and EGF (I + F + E) or together IGF1, FGF2 and VEGF_0.5 (I + F + V). Bright field images of 3D Matrigel (upper), maximum intensity projections of tiled confocal z-stack images of 3D Matrigel (middle), maximum intensity projections of z-stack confocal images (lower). Staining for endothelial marker Ulex Europaeus Agglutinin I (UEA, green), actin stained with phalloidin rhodamine (red) and nuclei with DAPI (blue). Scale bar is 2 mm (upper), 1 mm (middle), 200 μm (lower). (**B**) Area covered with 3D Matrigel during the course of 7 days in EGM2 (n = 16), EGM2-GFs supplemented with IGF1 (n = 4), FGF2 (n = 2), EGF (n = 2), I + F (n = 3), I + F + E (n = 3) and I + F + V (n = 3). Data are shown as mean ± SD. (**C**) Total length of formed HUVEC network in EGM2 (n = 69), EGM2-GFs (n = 48), EGM2-GFs supplemented with IGF1 (n = 13), FGF2 (n = 15), EGF (n = 16), I + F (n = 16), I + F + E (n = 19) and I + F + V (n = 16). Data are shown as Tukey-style boxplots with the median, IQR, whiskers (1.5 × IQR) and outliers. Each of the tested media was compared to EGM2 and Mann Whitney test was used for statistical analysis. ** p < 0.01, *** p < 0.001, **** p < 0.0001.

Subsequently, we proceeded to assess the effects of individual GFs found in complete EGM2 medium (IGF1, FGF2, EGF, and VEGF) on DPSC-induced Matrigel remodeling and HUVEC network formation. VEGF is commonly used in hydrogel vascularization protocols^29,30^. We initially examined the utilization of 0.5 ng/ml VEGF as a single growth factor in the medium. This concentration is present in complete EGM2, but it did not improve DPSC elongation or HUVEC network formation (Fig. 3A–C). Increasing the concentration to 10 ng/ml resulted in the longer HUVEC sprouts, but overall, it did not lead to the extensive enhancement in vascularization. Further elevating of VEGF concentration to 50 and 100 ng/ml led to an increase in HUVEC network length compared to 0.5 ng/ml VEGF, although the values were still significantly lower than in the complete EGM2 (Fig. 3C). Consequently, we focused on the other three GFs from the complete EGM2 (Fig. 4A–C). Supplementing with IGF1 mainly caused bulk grouping of DPSCs (Fig. 4A). This may explain why the addition of IGF1 resulted in more pronounced Matrigel area reduction compared to FGF2 or EGF (Fig. 4B). The network length was slightly longer when IGF1 or FGF2 were used individually compared to EGF (Fig. 4C). Thus, we decided to investigate the combined potency of IGF1 and FGF2 (I + F), as well as addition of EGF or 0.5 ng/ml VEGF (I + F + E and I + F + V, respectively). Medium with I + F led to the more pronounced Matrigel remodeling as shown by an area reduction to 32.77 ± 7.84% compared to individual factors (55.26 ± 19.24% IGF1 alone and 74.02 ± 0.02% FGF2 alone), as well as increase in network length compared to the individual factors (Fig. 4B,C). Although the network length remained significantly lower than in complete EGM2, the hydrogel remodeling with those two GFs was comparable to that observed in complete EGM2 medium. Addition of EGF or VEGF to I + F medium did not lead to further changes in the analyzed parameters (Fig. 4A–C).

### Addition of IGF1 and FGF2 to the less inductive MSC medium can improve the vascularization in 3D hydrogels

**Fig. 5:**
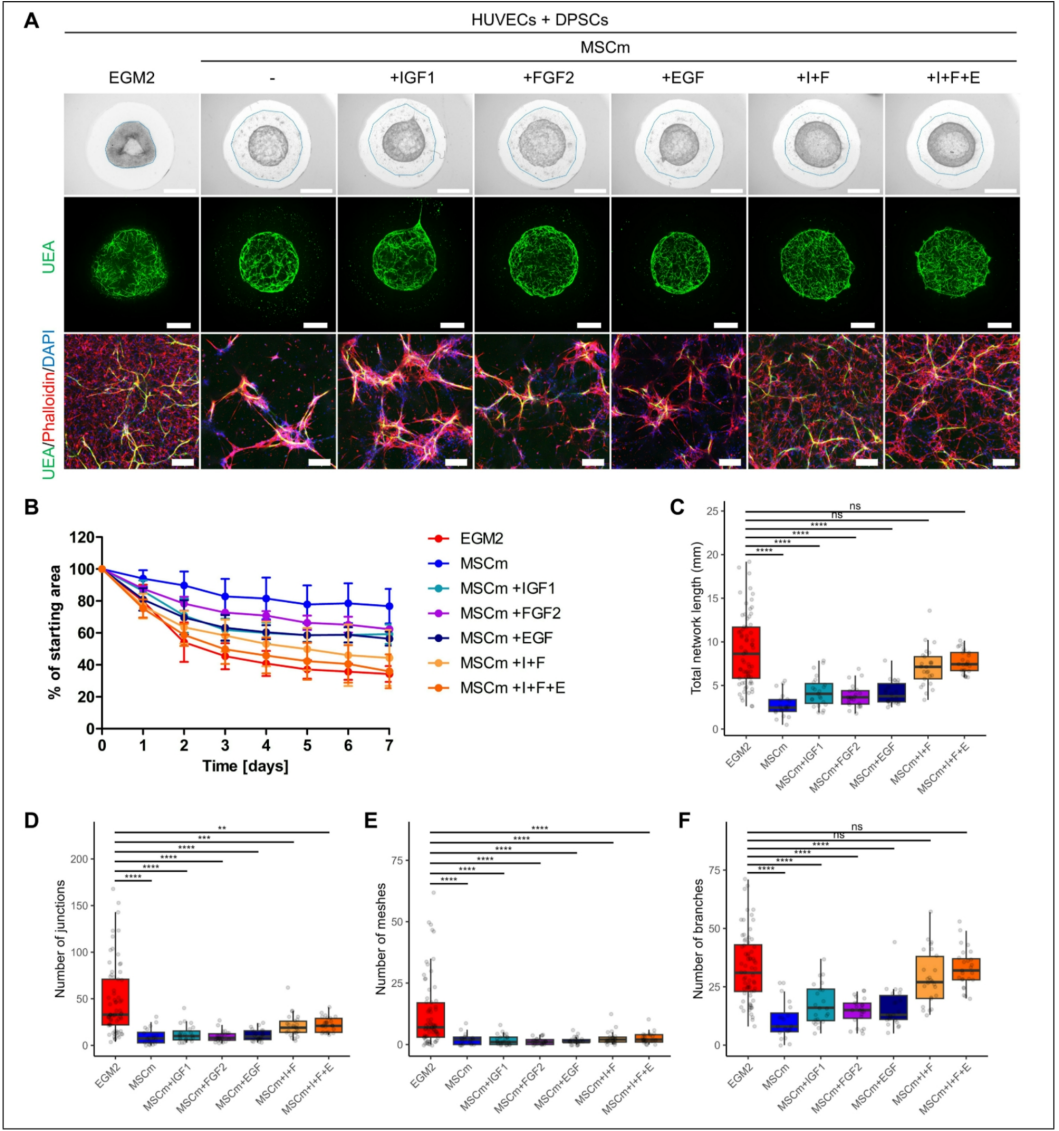
Addition of growth factors IGF1 and FGF2 to MSC medium can improve HUVEC network formation in co-cultures with DPSCs in 3D Matrigel. (**A**) HUVEC spheroids in co-culture with DPSCs at day 7 of cultivation in media: EGM2, MSC medium (MSCm), MSC medium supplemented with IGF1, FGF2, EGF, IGF1 and FGF2 (I + F) or IGF1, FGF2 and EGF (I + F + E). Bright field images of 3D Matrigel (upper), maximum intensity projections of tiled confocal z-stack images of 3D Matrigel (middle), maximum intensity projections of z-stack confocal images (lower). Staining for endothelial marker Ulex Europaeus Agglutinin I (UEA, green), actin stained with phalloidin rhodamine (red) and nuclei with DAPI (blue). Scale bar is 2 mm (upper), 1 mm (middle), 200 μm (lower). (**B**) Area covered with 3D Matrigel during the course of 7 days shown as percentage of starting area. Samples in media: EGM2 (n = 16), MSC medium (n = 5), MSC medium with IGF1 (n = 3), MSC medium with FGF2 (n = 3), MSC medium with EGF (n = 3), MSC medium with IGF1 and FGF2 (I + F, n = 3), MSC medium with IGF1, FGF2 and EGF (I + F + E, n = 4). Data are shown as mean ± SD. (**C**) Total length of formed HUVEC network, (**D**) number of junctions, (**E**) number of meshes and (**F**) number of branches in media: EGM2 (n = 69), MSC medium (n = 20), MSC medium with IGF1 (n = 23), MSC medium with FGF2 (n = 23), MSC medium with EGF (n = 22), MSC medium with IGF1 and FGF2 (n = 25), MSC medium with IGF1, FGF2 and EGF (n = 25). Data are shown as Tukey-style boxplots with the median, IQR, whiskers (1.5 × IQR) and outliers. Each of the tested media was compared to EGM2 and Mann Whitney test was used for statistical analysis. ** p < 0.01, *** p < 0.001, **** p < 0.0001.

Based on our results showing that specific GFs are the important drivers of HUVEC network formation in co-culture with DPSCs in Matrigel, we decided to test whether enrichment with these GFs could ensure vascularization also in other types of media that are not primarily used for culturing of ECs, such as MSC medium. The addition of individual GFs to MSC medium led to the clustering of DPSCs around HUVEC spheroids to unevenly distribute them in Matrigel (Fig. 5A). We observed a slight shrinkage of the hydrogel cylinder, but HUVECs expanded minimally and the length of the endothelial network compared to growth in MSC medium was significantly lower than in EGM2 (Fig. 5B,C). Positive effects on DPSC distribution and endothelial network formation were obtained by combining IGF1 and FGF2 (I + F) or adding EGF (I + F + E). Correspondingly, we observed a decrease in the area covered with Matrigel close to value as for full EGM2. Analysis of additional network parameters number of junctions, number of meshes, and number of branches, showed that the observed increase in the total network length upon addition of combinations of GFs was mainly due to the increase in number of branches while the number of junctions and number of meshes stayed significantly lower than in EGM2 (Fig. 5D–F).

To confirm that the inductive effect of these GFs is not specifically related to the used hydrogel, we evaluated the HUVEC network formation in metacrylated gelatin (GelMA) (Fig. S5). Also, in GelMA, a supportive effect of the combination of these GFs in the MSC medium on the length of the endothelial network and the uniform distribution of SCs was apparent, as observed in Matrigel. These results suggest that the combination of GFs is a key inducer of network formation, usable in a variety of setups with various media and materials.

### Co-culture of HUVECs and ASCs undergoes similar inductive effect of growth factors IGF1, FGF2, and EGF

**Fig. 6:**
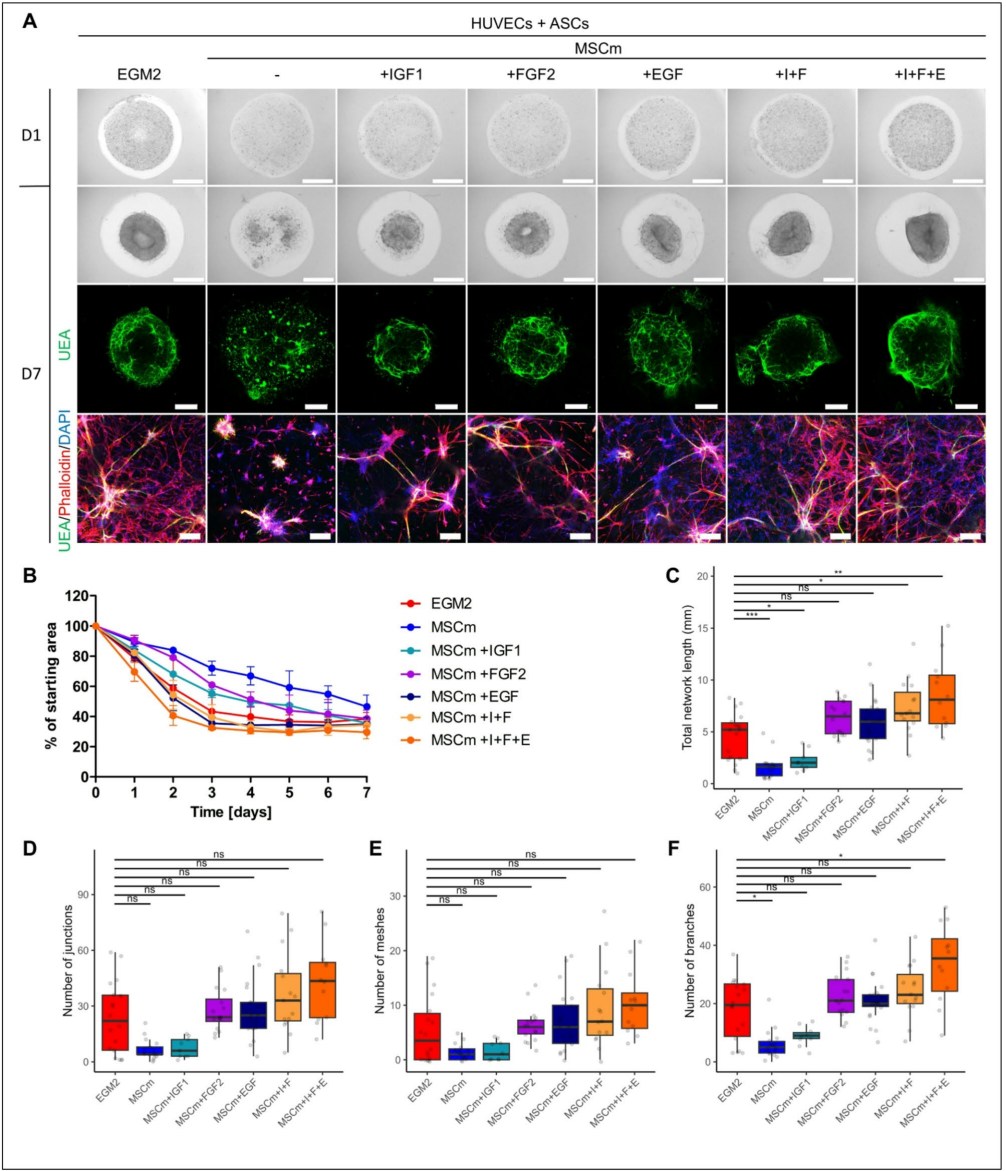
HUVEC network formation in co-culture with ASCs and effect of GFs in 3D Matrigel. (**A**) HUVECs in the form of spheroids with DPSCs at day 1 (D1) and day 7 (D7) of cultivation in media: EGM2, MSC medium (MSCm), MSC medium supplemented with IGF1, FGF2, EGF, IGF1 and FGF2 (I + F) or IGF1, FGF2 and EGF (I + F + E). Bright field images of Matrigel (upper), maximum intensity projections of tiled confocal z-stack images of 3D Matrigel (middle), maximum intensity projections of z-stack confocal images (lower). Staining for endothelial marker Ulex Europaeus Agglutinin I (UEA, green), actin stained with phalloidin rhodamine (red) and nuclei with DAPI (blue). Scale bar is 2 mm (upper), 1 mm (middle), 200 μm (lower). (**B**) Area covered with 3D Matrigel during the course of 7 days shown as percentage of starting area. Samples in media: EGM2 (n = 4), MSC medium (MSCm, n = 2), MSC medium with IGF1 (n = 2), MSC medium with FGF2 (n = 2), MSC medium with EGF (n = 2), MSC medium with IGF1 and FGF2 (I + F, n = 2), MSC medium with IGF1, FGF2 and EGF (I + F + E, n = 2). Data are shown as mean ± SD. (**C**) Total length of formed HUVEC network, (**D**) number of junctions, (**E**) number of meshes and (**F**) number of branches in media: EGM2 (n = 18), MSC medium (n = 17), MSC medium with IGF1 (n = 9), MSC medium with FGF2 (n = 16), MSC medium with EGF (n = 21), MSC medium with IGF1 and FGF2 (I + F, n = 15), MSC medium with IGF1, FGF2 and EGF (I + F + E, n = 12). Data are shown as Tukey-style boxplots with the median, IQR, whiskers (1.5 × IQR) and outliers. Each of the tested media was compared to EGM2 and Mann Whitney test was used for statistical analysis. * p < 0.05, ** p < 0.01, *** p < 0.001.

In generation of 3D vascularized constructs, ASCs are another cell type that is extensively used due to their easy accessibility and isolation from lipoaspirates^9,25,31^. Comparing the results with DPSCs, we noticed that ASCs have the ability to induce 3D network formation and remodeling of Matrigel to a similar degree as DPSCs in EGM2 medium; however, when using cell concentrations 2 × 10^6^ and 4.5 × 10^6^ ASCs/ml, a significantly shorter HUVEC network was measured in contrast to the same concentrations of DPSCs (Fig. S6). When the effects of MSC medium supplementation with selected GFs (IGF1, FGF2, and EGF) were tested on HUVEC and ASC co-cultures, we observed more pronounced shrinkage of Matrigel cylinders compared to MSC medium (Fig. 6A,B). EGF and combination of I + F and I + F + E showed the strongest effect, inducing the area reduction to the similar extent as EGM2, whereas the effects of IGF1 and FGF2 were more moderate. Regarding the length of vascular network, FGF2 and EGF when applied alone induced increase in network length compared to MSC medium or MSC medium with IGF1 (Fig. 6C). The same effect could be observed when using combinations of GFs (I + F and I + F + E) for supplementation of MSC medium. Increase in network length under the conditions where MSC medium was supplemented with FGF2, EGF, I + F or I + F + E was complemented with the increase in other network parameters - the number of junctions, number of meshes, and number of branches compared to MSC medium alone (Fig. 6D–F). Although there could be seen some differences in the effect of individual GFs when DPSCs or ASCs were used, the overall conclusion is that supplementation of MSC medium with GFs could improve HUVEC vascularization of Matrigel regardless of the SC type that we tested.

### IGF1, FGF2, and EGF can induce the HUVEC network formation under the xeno-free and chemically defined conditions

**Fig. 7:**
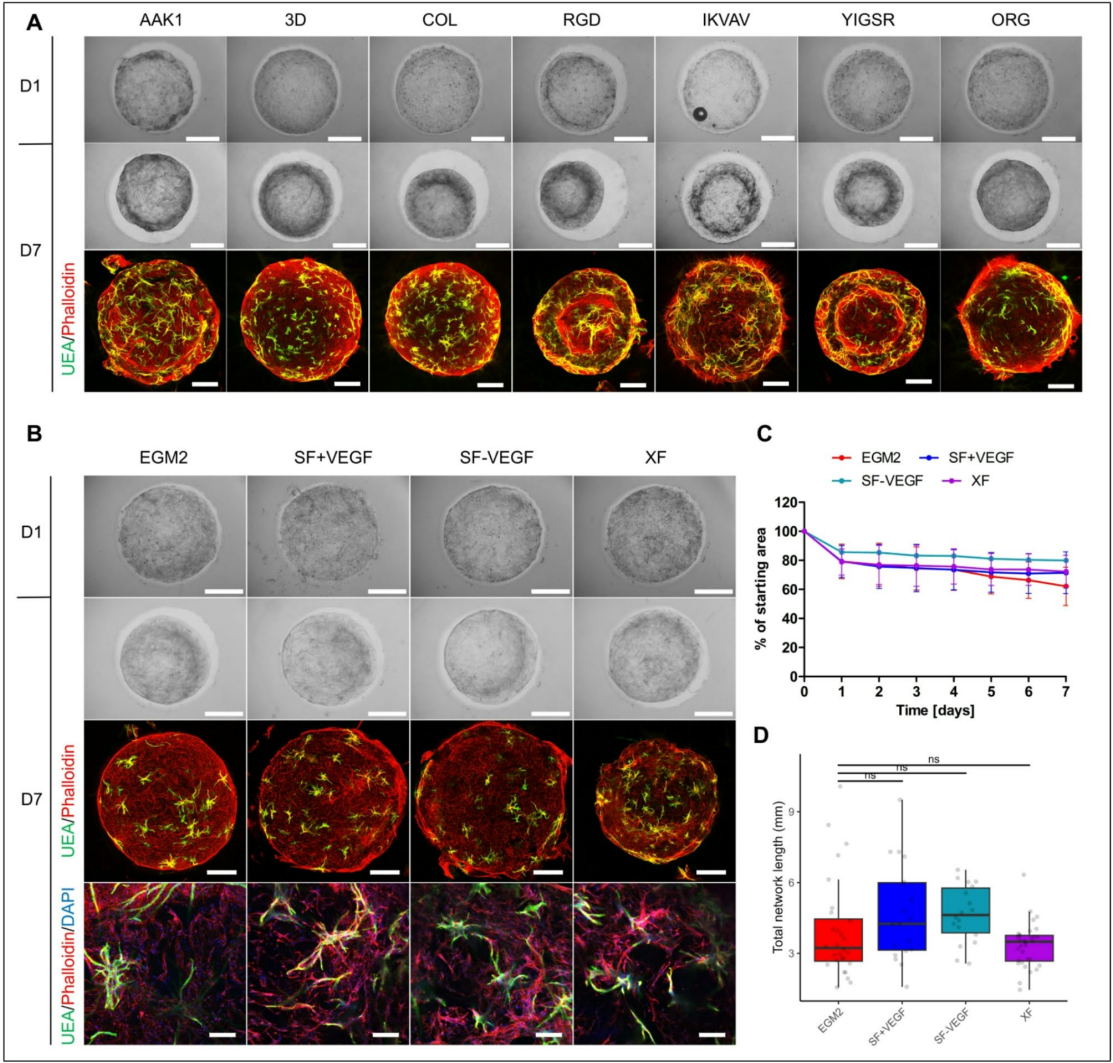
HUVEC vascular network formation in serum- and xeno-free conditions. (**A**) Bright field images showing HUVEC network formation in co-culture with DPSCs in EGM2 in VitroGel HC hydrogels: AAK1, 3D, COL, RGD, IKVAV, YIGSR and ORG at day 1 and day 7 of culture (D1 and D7, respectively). Maximum intensity projections of z-stack confocal images showing HUVEC network stained with UEA (green) and actin stained with phalloidin rhodamine (red). Scale bars are 2 mm on the bright field images and 1 mm on the maximum intensity projection images showing the whole hydrogel. (**B**) HUVEC network formation in co-culture with DPSCs in VitroGel HC COL hydrogel. Used media are EGM2 (control), EGM2 with FCS substituted with B27 (serum free with VEGF, SF + VEGF), EGM2 with FCS substituted with B27 and without VEGF (serum-free without VEGF, SF-VEGF), and EGM2 medium without FCS and VEGF (xeno-free, XF). Scale bar is 2 mm on the bright field images, 1 mm on the maximum intensity projection images showing the whole hydrogel, and 200 μm on the images showing a detail. (**C**) Change of area covered with VitroGel COL hydrogel in media: EGM2 (n = 2), SF + VEGF (n = 2), SF-VEGF (n = 1), and XF (n = 2) during the course of 7 days. Data are presented as percentage of the starting area and shown as mean ± SD. (**D**) Total length of HUVEC network in VitroGel COL hydrogel in the media: EGM2 (n = 28), SF + VEGF (n = 19), SF-VEGF (n = 18), and XF (n = 32). Data are shown as Tukey-style boxplots with the median, IQR, whiskers (1.5 × IQR) and outliers. Each of the tested media was compared to EGM2 and Mann Whitney test was used for statistical analysis.

In the creation of clinically relevant vascularized tissue constructs, the limiting factors of media and hydrogels are their animal and chemically undefined origin. This motivated us to investigate HUVEC network formation in co-cultures with DPSCs in commercially available xeno-free VitroGel hydrogels (Fig. 7A). We utilized seven different xeno-free hydrogels which supported cell survival and endothelial network formation. Although the AAK1 (angiogenesis assay kit) hydrogel supported the HUVEC network formation, polymerization of this hydrogel occurred in the order of seconds and handling of the gel was difficult. When diluted, AAK1 did not support the HUVEC sprouting (Fig. S7). On the contrary, COL hydrogel was easy to manipulate in the tested dilution and thus was used for the subsequent experiments. Furthermore, the aim was to replace FCS in EGM2 with a chemically defined B27 supplement, and we labeled this medium as serum-free (SF) and tested a VEGF-free variant (SF-VEGF). Although this medium was serum-free in composition, it still contained the animal product bovine serum albumin (BSA) as part of the B27 supplement. Thus, we excluded also B27 as well as FCS and VEGF, which we labeled as xeno-free medium (XF). When these media were applied to HUVECs in co-culture with DPSCs in Matrigel, we observed a reduction in the area covered with hydrogel to a similar extent as for full EGM2, although all three media were less inducible in terms of network formation (Fig. S8). In a xeno-free COL hydrogel (Fig. 7B–D), after initial shrinkage, we observed slight change in hydrogel size during 7 days regardless of the media tested (Fig. 7B,C). SF and XF media promoted HUVEC sprouting in the COL hydrogel to the similar extent as EGM2 (Fig. 7D). All tested conditions, including the xeno-free one, however allowed spreading of ECs from spheroids and matrix remodeling, key steps allowing vascularization using this approach.

## Discussion

In this study, we developed a 3D vascular system in GFR-Matrigel using endothelial cells (HUVECs) and stromal cells (DPSCs or ASCs). Despite Matrigel’s drawbacks, including its animal origin and batch variability^32^, it remains integral to many human pluripotent stem cell differentiation protocols^2,3,33^. To address the nutrient limitations associated with long-term cultures of human pluripotent stem cell-derived organoids, we focused on formation of endothelial network within Matrigel. We developed a system, which precisely defines hydrogel shape within an agarose ring, providing enhanced experimental reproducibility. This method differed from the more typical approach of creating hemispherical domes on a thin Matrigel layer in a well. By introducing this cylindrical setup, we ensured a consistent starting size and shape for the hydrogel, with the cells evenly distributed throughout the hydrogel. This design allowed us to assess the vascular network formation and track physical changes in hydrogel cylinders during cell culture. To assess EC network formation, a 2D tube formation assay on Matrigel layer is commonly used^4^. On the other hand, 3D Matrigel was shown not to be a suitable choice for vascularization because of the inability of ECs to migrate through the hydrogel and remodel it due to its stiffness and/or composition^34^. Even the hybrid hydrogels of Matrigel and collagen type I did not support network formation when HUVECs were seeded alone^35^. Preferable scaffolds for 3D vascular network formation are hydrogels such as fibrin^6,7,36^, collagen^37,38^, and hybrid hydrogels^9,39^. ECs, seeded on gelatin-coated microcarriers and embedded in fibrin hydrogels, can migrate and form capillary-like structures in the presence of angiogenic factors fibronectin, FGF2, and VEGF^36^. Also, when ECs were seeded as single cells within fibrin hydrogels, they formed the tubular structures under stimulation with FGF2 and VEGF without addition of SCs^40,41^. We showed that HUVECs without SCs in Matrigel cannot form the network when seeded neither as spheroids nor single cells regardless of the supportive GFs present in the medium. Although DPSCs are known for their pro-angiogenic secretome^17,18^, we showed that DPSC-conditioned medium cannot help to overcome this problem in Matrigel. The efficiency of SC-mediated HUVEC vascularization in our setup was dependent on the SC concentration inside the hydrogel. After 7 days, hydrogels with low numbers of seeded SCs were less remodeled and had a less developed EC network. In contrast, Margolis and colleagues demonstrated that regardless of the initial number of SCs and ECs in the fibrin gel, the total number of cells equilibrated between the conditions after 7 days^10^. As other studies on ECs are performed in fibrin with SCs seeded on the top of hydrogels^42^, this supports that it is the fibrous nature of hydrogels which guides formation of EC network, making fibrin or collagen frequently preferred over Matrigel.

ASCs have been used in many studies focused on vascularization of different hydrogels in co-cultures with ECs, confirming their pro-angiogenic activity^31,35,39,43^. We showed that DPSCs, although leading to Matrigel remodeling to the similar extent as ASCs, supported formation of longer EC networks than ASCs. Our gene expression analysis further supports these observations by showing that stromal–endothelial co-cultures upregulated *CXCL12*, *VEGFA*, *VWF*, and *MMP2* over time. The induction of CXCL12 is consistent with IGF1–CXCR4 crosstalk previously described in mesenchymal stromal cells^44^, suggesting that IGF1 stimulation may enhance angiogenesis indirectly through stromal signaling. Increased *VEGFA* and *VWF* expression indicate angiogenic activation and endothelial maturation, while *MMP2* upregulation points to matrix remodeling as a key process enabling network expansion. Together, these molecular changes align with our morphological findings and highlight how SCs orchestrate both paracrine signaling and ECM modification to support endothelial network formation in 3D Matrigel.

Studying the interactions of different cell types in 3D is challenging because of the different culture conditions required for survival and function of each cell type. Several groups put efforts into defining the medium that would support EC network formation in collagen^38^, fibrin^6^, and hybrid collagen-Matrigel hydrogels^9^, as the purpose of commercially available EGM2 medium is primarily propagation of ECs. When it comes to co-cultures of ECs with SCs, researchers usually use a simple approach of mixing culture media of each cell type^22,45^ We, on the other hand, wanted to take a closer look at components of EGM2 medium which positively influence the vascularization process. Moreover, DPSCs that we used are multipotent stem cells, and their differentiation is tightly regulated by the medium composition^15^. Luzuriaga and colleagues used neurogenic medium in their study that led to expression of CD31 in DPSCs^46^. We showed that DPSCs did not express the endothelial markers when cultured in EGM2 or MSC medium and thus did not contribute to the EC network in this way. We also showed that the combination of low concentration of FCS and hydrocortisone may have an inhibitory effect on remodeling of hydrogel by DPSCs in our 3D model. Inhibitory effect of hydrocortisone could be, in part, due to abolishment of VEGF expression in mural cells^47^. On the other hand, heparin is known for stabilization of GFs and can augment their effects^48^. However, the early works on ECs in fibrin gels showed the inhibitory effect of hydrocortisone and heparin on FGF2-stimulated angiogenesis^36^. All these results indicate that the effect of every individual component of the 3D model is highly dependent on the complex interactions involving the cells, ECM, and medium components.

DPSCs and ASCs share common properties with other mesenchymal stem cells^49,50^. One of the characteristics of mesenchymal cells is their ability to cluster towards a single center when placed on Matrigel in a process termed self-condensation or collective condensation^51^. Based on our results, DPSCs and ASCs can perform the same when embedded in Matrigel placed on the top of cell-free Matrigel layer. Published data from various groups suggest that IGF1 affects proliferation and differentiation of DPSCs^52–54^. However, there are no data on its effect on self-condensation of mesenchymal stem cells which seems to be a process based primarily on local cell movements rather than locally altered proliferation rates^55^. Based on the effects of IGF1 on shrinking of Matrigel in our setup, it gives us some insights on potential role of IGF1 on the process of collective condensation, although, this should be further investigated. Delle Monache et al. showed that the presence of FGF2 in EGM2 medium is a major factor for the long-term stabilization of endothelial tubes by DPSCs in 2D assay on Matrigel^28^. The same study showed that culture of DPSCs in EGM2 could stimulate the acquisition of pericyte-like phenotype in DPSC population. On the contrary, DPSCs injected intramyocardially in nude rats did not differentiate into neither smooth muscle cells nor ECs confirming the importance of microenvironment in guiding the cell differentiation^56^. VEGF is a well-known potent pro-angiogenic factor that is crucial for establishment of vascular network during embryo development^29^. Some publications show the synergetic effect of FGF2 and VEGF on vascular network formation in collagen and fibrin hydrogels^40,57^. However, Davis and colleagues showed that VEGF could prime ECs and facilitate their responsiveness to pro-angiogenic factors: stem cell factor (SCF), interleukin-3 (IL-3), stromal-derived factor-1a (SDF-1a), FGF2, and insulin rather than being sufficient to induce vascularization^8^. It is also known that a gradient of VEGF is more important than the presence of VEGF per se^58,59^, which may explain why an increased VEGF concentration did not show more pronounced inductive effect under static conditions in our study. FGF2 as an upstream of VEGF signaling can induce the VEGF production in ECs and SCs^60–62^. In this way, it can be expected that FGF2 induces more localized VEGF production by SCs that can form local VEGF gradient and induce angiogenesis in our model. Besides this effect, FGF2 can directly induce DPSCs’ proliferation and migration^52,63^.

In our Matrigel-based system we did not observe clear lumenized structures, which limited the possibility of conducting perfusion or other functional assays. This limitation is consistent with previous reports highlighting the mechanical constraints of pure Matrigel in supporting stable vessel morphogenesis, where networks rapidly regress or collapse under flow conditions^12^. For this reason, our study was focused on defining the minimal medium requirements and stromal cell contributions necessary to initiate 3D endothelial network formation.

In recent years, the efforts have been put in development of chemically defined and xeno-free conditions for culture of human pluripotent stem cells and development of in vitro models^64,65^. We employed the xeno-free VitroGel hydrogels and showed that, together with chemically-defined or xeno-free media, can support the ECs sprouting in co-cultures with SCs. In this study, experiments with xeno-free hydrogels were included primarily to confirm that the GF requirements identified in Matrigel also apply under translationally relevant conditions. While these findings are encouraging, and consistent across multiple VitroGel variants, they remain preliminary. Longer culture periods, functional validation including perfusion assays, and direct comparisons with Matrigel will be important directions for future work.

In summary, we established a model that allows systematic monitoring of stromal–endothelial interactions and the impact of specific GFs in 3D hydrogels. Our findings indicate that VEGF alone is not sufficient to support robust vascularization in this context, though high concentrations provided partial benefits. Instead, stromal cell-mediated remodeling combined with stimulation by IGF1, FGF2, and EGF emerged as the primary drivers of vascular network formation, even under serum-free and chemically defined conditions. These insights may guide the development of more physiologically relevant and translationally applicable vascularized tissue models, including organoid co-cultures.

## Supplementary Information

Below is the link to the electronic supplementary material.

Supplementary Material 1

## Data Availability

All data supporting the findings of this study are available within the article and its Supplementary Information files. Additional raw data are available from the corresponding author upon reasonable request.

## References

[1] Dellaquila, A., Le Bao, C. & Letourneur, D. Simon-Yarza, T. In vitro strategies to vascularize 3D physiologically relevant models. *Adv. Sci. (Weinh)*. **8**, e2100798 (2021). 34351702 10.1002/advs.202100798 PMC8498873

[2] Hocevar, S. E., Liu, L. & Duncan, R. K. Matrigel is required for efficient differentiation of isolated, stem cell-derived otic vesicles into inner ear organoids. *Stem Cell. Res.* **53**, 102295 (2021). 33773390 10.1016/j.scr.2021.102295 PMC8360351

[3] Han, L. et al. Generation of human embryonic stem cell-derived lung organoids. *STAR. Protoc.* **3**, 101270 (2022). 35403011 10.1016/j.xpro.2022.101270 PMC8987391

[4] Arnaoutova, I., George, J., Kleinman, H. K. & Benton, G. The endothelial cell tube formation assay on basement membrane turns 20: state of the science and the Art. *Angiogenesis* **12**, 267–274 (2009). 19399631 10.1007/s10456-009-9146-4

[5] Rao, R. R., Peterson, A. W., Ceccarelli, J., Putnam, A. J. & Stegemann, J. P. Matrix composition regulates three-dimensional network formation by endothelial cells and mesenchymal stem cells in collagen/fibrin materials. *Angiogenesis* **15**, 253–264 (2012). 22382584 10.1007/s10456-012-9257-1 PMC3756314

[6] Smith, A. O., Bowers, S. L. K., Stratman, A. N. & Davis, G. E. Hematopoietic stem cell cytokines and fibroblast growth factor-2 stimulate human endothelial cell-pericyte tube co-assembly in 3D fibrin matrices under serum-free defined conditions. *PLoS One*. **8**, e85147 (2013). 24391990 10.1371/journal.pone.0085147 PMC3877341

[7] Morin, K. T. & Tranquillo, R. T. In vitro models of angiogenesis and vasculogenesis in fibrin gel. *Exp. Cell. Res.* **319**, 2409–2417 (2013). 23800466 10.1016/j.yexcr.2013.06.006 PMC3919069

[8] Davis, G. E., Norden, P. R. & Bowers, S. L. K. Molecular control of capillary morphogenesis and maturation by recognition and remodeling of the extracellular matrix: functional roles of endothelial cells and pericytes in health and disease. *Connect. Tissue Res.* **56**, 392–402 (2015). 26305158 10.3109/03008207.2015.1066781 PMC4765926

[9] Andrée, B. et al. Formation of three-dimensional tubular endothelial cell networks under defined serum-free cell culture conditions in human collagen hydrogels. *Sci. Rep.* **9**, 5437 (2019). 30932006 10.1038/s41598-019-41985-6 PMC6443732

[10] Margolis, E. A. et al. Stromal cell identity modulates vascular morphogenesis in a microvasculature-on-a-chip platform. *Lab. Chip*. **21**, 1150–1163 (2021). 33538719 10.1039/d0lc01092h PMC7990720

[11] Rajasekar, S. et al. IFlowPlate-A customized 384-Well plate for the culture of perfusable vascularized colon organoids. *Adv. Mater.* **32**, e2002974 (2020). 33000879 10.1002/adma.202002974

[12] Kwak, T. J. & Lee, E. In vitro modeling of solid tumor interactions with perfused blood vessels. *Sci. Rep.* **10**, 20142 (2020). 33214583 10.1038/s41598-020-77180-1 PMC7677310

[13] Grainger, S. J., Carrion, B., Ceccarelli, J. & Putnam, A. J. Stromal cell identity influences the in vivo functionality of engineered capillary networks formed by co-delivery of endothelial cells and stromal cells. *Tissue Eng. Part. A*. **19**, 1209–1222 (2013). 23216113 10.1089/ten.tea.2012.0281 PMC3609639

[14] Carmeliet, P. Mechanisms of angiogenesis and arteriogenesis. *Nat. Med.* **6**, 389–395 (2000). 10742145 10.1038/74651

[15] Mattei, V. et al. Regenerative potential of DPSCs and revascularization: Direct, paracrine or autocrine effect? *Stem Cell. Rev. Rep.* **17**, 1635–1646 (2021). 33829353 10.1007/s12015-021-10162-6 PMC8553678

[16] Si, Z. et al. Adipose-derived stem cells: Sources, potency, and implications for regenerative therapies. *Biomed. Pharmacother*. **114**, 108765 (2019). 30921703 10.1016/j.biopha.2019.108765

[17] Bronckaers, A. et al. Angiogenic properties of human dental pulp stem cells. *PLoS One*. **8**, e71104 (2013). 23951091 10.1371/journal.pone.0071104 PMC3737205

[18] Gharaei, M. A., Xue, Y., Mustafa, K., Lie, S. A. & Fristad, I. Human dental pulp stromal cell conditioned medium alters endothelial cell behavior. *Stem Cell. Res. Ther.* **9**, 69 (2018). 29562913 10.1186/s13287-018-0815-3 PMC5861606

[19] Silveira, B. M. et al. Secretome from human adipose-derived mesenchymal stem cells promotes blood vessel formation and pericyte coverage in experimental skin repair. *PLoS One*. **17**, e0277863 (2022). 36534643 10.1371/journal.pone.0277863 PMC9762598

[20] Winkel, A. et al. Cell culture media notably influence properties of human mesenchymal stroma/stem-like cells from different tissues. *Cytotherapy* **22**, 653–668 (2020). 32855067 10.1016/j.jcyt.2020.07.005

[21] Leopold, B. et al. Outgrowth, proliferation, viability, angiogenesis and phenotype of primary human endothelial cells in different purchasable endothelial culture media: feed wisely. *Histochem. Cell. Biol.* **152**, 377–390 (2019). 31541300 10.1007/s00418-019-01815-2 PMC6842357

[22] Dissanayaka, W. L. et al. Coculture of dental pulp stem cells with endothelial cells enhances osteo-/odontogenic and angiogenic potential in vitro. *J. Endod*. **38**, 454–463 (2012). 22414829 10.1016/j.joen.2011.12.024

[23] Rosenstein, J. M., Mani, N., Silverman, W. F. & Krum, J. M. Patterns of brain angiogenesis after vascular endothelial growth factor administration in vitro and in vivo. *Proc. Natl. Acad. Sci. U S A*. **95**, 7086–7091 (1998). 9618543 10.1073/pnas.95.12.7086 PMC22748

[24] Silva, E. A. & Mooney, D. J. Effects of VEGF Temporal and Spatial presentation on angiogenesis. *Biomaterials* **31**, 1235–1241 (2010). 19906422 10.1016/j.biomaterials.2009.10.052 PMC2813952

[25] Streit, L. et al. A comprehensive in vitro comparison of Preparation techniques for fat grafting. *Plast. Reconstr. Surg.* **139**, 670e–682e (2017). 28234835 10.1097/PRS.0000000000003124

[26] Ying, G. L. et al. Aqueous Two-Phase emulsion Bioink-Enabled 3D Bioprinting of porous hydrogels. *Adv. Mater.* **30**, e1805460 (2018). 30345555 10.1002/adma.201805460 PMC6402588

[27] Carpentier, G. et al. Angiogenesis analyzer for ImageJ - A comparative morphometric analysis of ‘Endothelial tube formation assay’ and ‘Fibrin bead assay’. *Sci. Rep.* **10**, 11568 (2020). 32665552 10.1038/s41598-020-67289-8 PMC7360583

[28] Delle Monache, S. et al. In vitro conditioning determines the capacity of dental pulp stem cells to function as Pericyte-Like cells. *Stem Cells Dev.* **28**, 695–706 (2019). 30887879 10.1089/scd.2018.0192

[29] Byrne, A. M., Bouchier-Hayes, D. J. & Harmey, J. H. Angiogenic and cell survival functions of vascular endothelial growth factor (VEGF). *J. Cell. Mol. Med.* **9**, 777–794 (2005). 16364190 10.1111/j.1582-4934.2005.tb00379.x PMC6740098

[30] Pauty, J. et al. A vascular endothelial growth Factor-Dependent sprouting angiogenesis assay based on an in vitro human blood vessel model for the study of Anti-Angiogenic drugs. *EBioMedicine* **27**, 225–236 (2018). 29289530 10.1016/j.ebiom.2017.12.014 PMC5828365

[31] Asano, Y. et al. Construction of transplantable artificial vascular tissue based on adipose tissue-derived mesenchymal stromal cells by a cell coating and cryopreservation technique. *Sci. Rep.* **11**, 17989 (2021). 34504254 10.1038/s41598-021-97547-2 PMC8429436

[32] Hughes, C. S., Postovit, L. M. & Lajoie, G. A. Matrigel: a complex protein mixture required for optimal growth of cell culture. *Proteomics* **10**, 1886–1890 (2010). 20162561 10.1002/pmic.200900758

[33] Lancaster, M. A. & Knoblich, J. A. Generation of cerebral organoids from human pluripotent stem cells. *Nat. Protoc.* **9**, 2329–2340 (2014). 25188634 10.1038/nprot.2014.158 PMC4160653

[34] Zhang, S., Wan, Z. & Kamm, R. D. Vascularized organoids on a chip: strategies for engineering organoids with functional vasculature. *Lab. Chip*. **21**, 473–488 (2021). 33480945 10.1039/d0lc01186j PMC8283929

[35] Manikowski, D. et al. Human adipose tissue-derived stromal cells in combination with exogenous stimuli facilitate three-dimensional network formation of human endothelial cells derived from various sources. *Vascul Pharmacol.* **106**, 28–36 (2018). 29452238 10.1016/j.vph.2018.02.003

[36] Nehls, V. & Drenckhahn, D. A novel, microcarrier-based in vitro assay for rapid and reliable quantification of three-dimensional cell migration and angiogenesis. *Microvasc Res.* **50**, 311–322 (1995). 8583947 10.1006/mvre.1995.1061

[37] Koh, W., Stratman, A. N., Sacharidou, A. & Davis, G. E. In vitro three dimensional collagen matrix models of endothelial lumen formation during vasculogenesis and angiogenesis. *Methods Enzymol.* **443**, 83–101 (2008). 18772012 10.1016/S0076-6879(08)02005-3

[38] Bowers, S. L. K. et al. Defining an upstream VEGF (Vascular endothelial growth Factor) priming signature for downstream Factor-Induced endothelial Cell-Pericyte tube network coassembly. *Arterioscler. Thromb. Vasc Biol.* **40**, 2891–2909 (2020). 33086871 10.1161/ATVBAHA.120.314517 PMC7939123

[39] Ichanti, H. et al. Characterization of tissue engineered endothelial cell networks in composite Collagen-Agarose hydrogels. *Gels* **6**, 27 (2020). 32899293 10.3390/gels6030027 PMC7559300

[40] Lafleur, M. A., Handsley, M. M., Knäuper, V., Murphy, G. & Edwards, D. R. Endothelial tubulogenesis within fibrin gels specifically requires the activity of membrane-type-matrix metalloproteinases (MT-MMPs). *J. Cell. Sci.* **115**, 3427–3438 (2002). 12154073 10.1242/jcs.115.17.3427

[41] Montaño, I. et al. Formation of human capillaries in vitro: the engineering of prevascularized matrices. *Tissue Eng. Part. A*. **16**, 269–282 (2010). 19702510 10.1089/ten.TEA.2008.0550

[42] Nakatsu, M. N. et al. Angiogenic sprouting and capillary lumen formation modeled by human umbilical vein endothelial cells (HUVEC) in fibrin gels: the role of fibroblasts and Angiopoietin-1. *Microvasc Res.* **66**, 102–112 (2003). 12935768 10.1016/s0026-2862(03)00045-1

[43] Rocha, L. A. et al. In vitro evaluation of ASCs and HUVECs Co-cultures in 3D biodegradable hydrogels on neurite outgrowth and vascular organization. *Front. Cell. Dev. Biol.* **8**, 489 (2020). 32612997 10.3389/fcell.2020.00489 PMC7308435

[44] Lee, H. T. et al. Role of IGF1R(+) MSCs in modulating neuroplasticity via CXCR4 cross-interaction. *Sci. Rep.* **6**, 32595 (2016). 27586516 10.1038/srep32595 PMC5009335

[45] Sierra-Parraga, J. M. et al. Reparative effect of mesenchymal stromal cells on endothelial cells after hypoxic and inflammatory injury. *Stem Cell. Res. Ther.* **11**, 352 (2020). 32787906 10.1186/s13287-020-01869-3 PMC7424997

[46] Luzuriaga, J. et al. Human dental pulp stem cells grown in neurogenic media differentiate into endothelial cells and promote neovasculogenesis in the mouse brain. *Front. Physiol.* **10**, 347 (2019). 30984027 10.3389/fphys.2019.00347 PMC6447688

[47] Nauck, M., Karakiulakis, G., Perruchoud, A. P., Papakonstantinou, E. & Roth, M. Corticosteroids inhibit the expression of the vascular endothelial growth factor gene in human vascular smooth muscle cells. *Eur. J. Pharmacol.* **341**, 309–315 (1998). 9543253 10.1016/s0014-2999(97)01464-7

[48] Folkman, J., Shing, Y. & Angiogenesis *J. Biol. Chem.* **267**, 10931–10934 (1992). 1375931

[49] Oh, M. & Nör, J. E. The perivascular niche and Self-Renewal of stem cells. *Front. Physiol.* **6**, 367 (2015). 26696901 10.3389/fphys.2015.00367 PMC4667083

[50] Minteer, D., Marra, K. G. & Rubin, J. P. Adipose-derived mesenchymal stem cells: biology and potential applications. *Adv. Biochem. Eng. Biotechnol.* **129**, 59–71 (2013). 22825719 10.1007/10_2012_146

[51] Takebe, T. et al. Vascularized and complex organ buds from diverse tissues via mesenchymal Cell-Driven condensation. *Cell. Stem Cell.* **16**, 556–565 (2015). 25891906 10.1016/j.stem.2015.03.004

[52] Kim, S. G. et al. Effects of growth factors on dental stem/progenitor cells. *Dent. Clin. North. Am.* **56**, 563–575 (2012). 22835538 10.1016/j.cden.2012.05.001 PMC4112411

[53] Lv, T. et al. Insulin-like growth factor 1 promotes the proliferation and committed differentiation of human dental pulp stem cells through MAPK pathways. *Arch. Oral Biol.* **72**, 116–123 (2016). 27573439 10.1016/j.archoralbio.2016.08.011

[54] Lu, W. et al. Effects of vascular endothelial growth factor and insulin growth factor–1 on proliferation, migration, osteogenesis and vascularization of human carious dental pulp stem cells. *Mol. Med. Rep.* **20**, 3924–3932 (2019). 31485628 10.3892/mmr.2019.10606

[55] Nimiritsky, P. P. et al. Unveiling mesenchymal stromal cells’ organizing function in regeneration. *Int. J. Mol. Sci.* **20**, 823 (2019). 30769851 10.3390/ijms20040823 PMC6413004

[56] Gandia, C. et al. Human dental pulp stem cells improve left ventricular function, induce angiogenesis, and reduce infarct size in rats with acute myocardial infarction. *Stem Cells*. **26**, 638–645 (2008). 18079433 10.1634/stemcells.2007-0484

[57] Stratman, A. N., Davis, M. J. & Davis, G. E. VEGF and FGF prime vascular tube morphogenesis and sprouting directed by hematopoietic stem cell cytokines. *Blood* **117**, 3709–3719 (2011). 21239704 10.1182/blood-2010-11-316752 PMC3083293

[58] Gerhardt, H. et al. VEGF guides angiogenic sprouting utilizing endothelial tip cell filopodia. *J. Cell. Biol.* **161**, 1163–1177 (2003). 12810700 10.1083/jcb.200302047 PMC2172999

[59] Ruhrberg, C. et al. Spatially restricted patterning cues provided by heparin-binding VEGF-A control blood vessel branching morphogenesis. *Genes Dev.* **16**, 2684–2698 (2002). 12381667 10.1101/gad.242002 PMC187458

[60] Claffey, K. P. et al. Fibroblast growth factor 2 activation of stromal cell vascular endothelial growth factor expression and angiogenesis. *Lab. Invest.* **81**, 61–75 (2001). 11204275 10.1038/labinvest.3780212

[61] Murakami, M. & Simons, M. Fibroblast growth factor regulation of neovascularization. *Curr. Opin. Hematol.* **15**, 215–220 (2008). 18391788 10.1097/MOH.0b013e3282f97d98 PMC2745288

[62] Yang, X. et al. Fibroblast growth factor signaling in the vasculature. *Curr. Atheroscler Rep.* **17**, 509 (2015). 25813213 10.1007/s11883-015-0509-6 PMC4593313

[63] Liu, K., Yu, S., Ye, L. & Gao, B. The regenerative potential of bFGF in dental pulp repair and regeneration. *Front. Pharmacol.* **12**, 680209 (2021). 34354584 10.3389/fphar.2021.680209 PMC8329335

[64] Cherne, M. D. et al. A synthetic Hydrogel, VitroGel^®^ ORGANOID-3, improves immune Cell-Epithelial interactions in a tissue chip Co-Culture model of human gastric organoids and dendritic cells. *Front. Pharmacol.* **12**, 707891 (2021). 34552484 10.3389/fphar.2021.707891 PMC8450338

[65] Zeiringer, S. et al. Development and characterization of an in vitro intestinal model including extracellular matrix and macrovascular endothelium. *Mol. Pharm.* **20**, 5173–5184 (2023). 37677739 10.1021/acs.molpharmaceut.3c00532 PMC10548470

