# Supplementary material for: 3D endothelial network formation in hydrogels improved by stromal cells and specific growth factors
Source: Sci Rep. 2025 Nov 24;15:41524. doi: 10.1038/s41598-025-25381-x (PMC12644823; doi:10.1038/s41598-025-25381-x)
Supplement: Supplementary file 1 — Supplementary Material 1 [file 41598_2025_25381_MOESM1_ESM.pdf]

Supplementary Information

to

3D endothelial network formation in hydrogels improved by stromal cells and specific growth factors

Ivana Acimovic[1], Vaclav Chochola[1], Jose Luis Herrera[3], Ales Hampl[1,2], Josef Jaros[1,2,*]

[1]Department of Histology and Embryology, Faculty of Medicine, Masaryk University; Brno, 62500, Czech Republic.

[2]Cell and Tissue Regeneration, International Clinical Research Center, St. Anne's University Hospital; Brno, 60200, Czech Republic.

[3]Division of Pulmonary, Critical Care and Sleep Medicine, University of Miami Miller School of Medicine, Miami, FL, USA

[*]Corresponding author – jaros.josef@hotmail.com

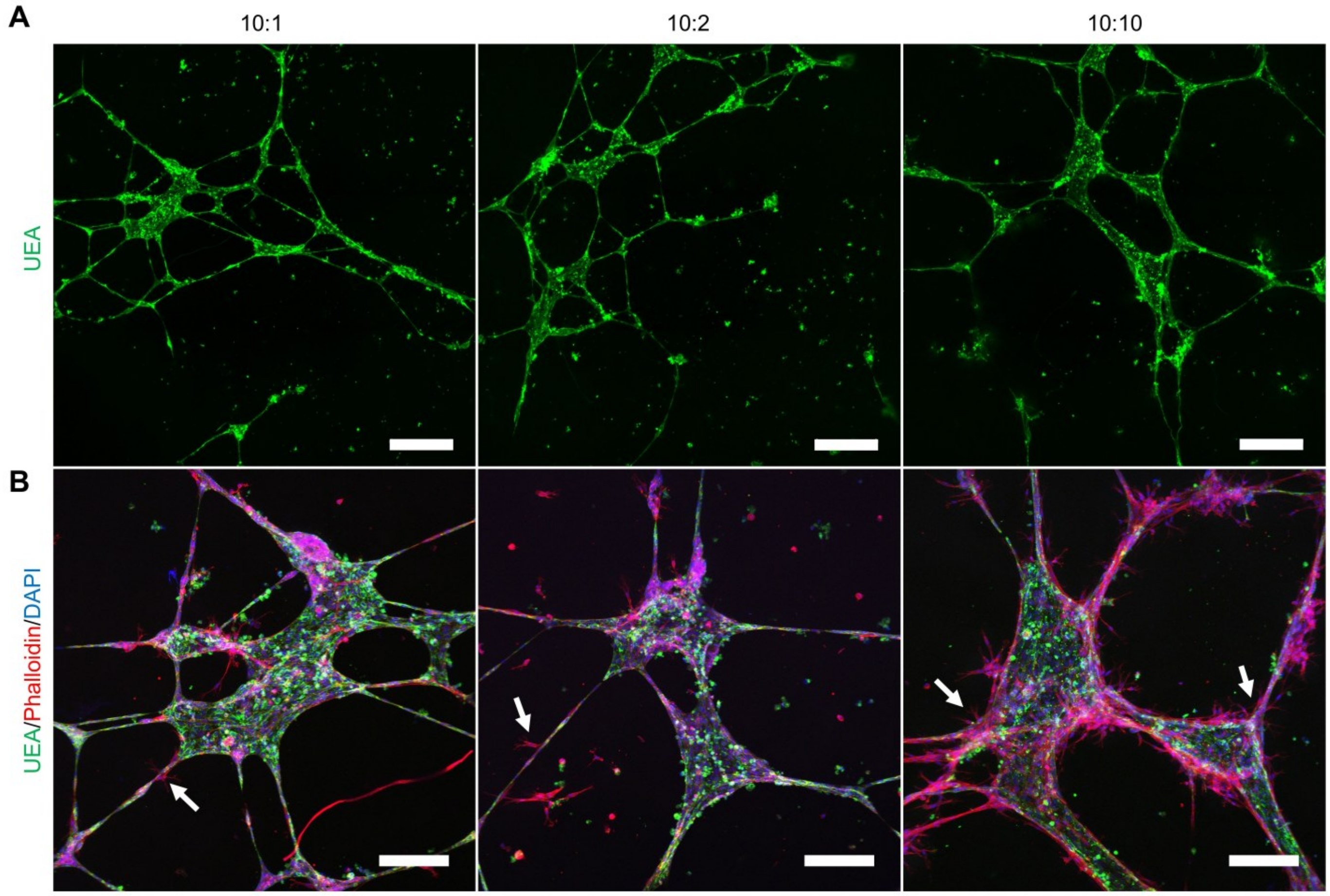

Figure S1: Stabilization of HUVEC tubular network by DPSCs on Matrigel in EGM2. (A) *HUVEC tubular network stained with UEA (green) 48 h after seeding of HUVECs. DPSCs (not stained) were added 24 h after HUVECs had been seeded. HUVECs : DPSCs ratios are 10:1, 10:2, and 10:10, respectively. Scale bar is 500 µm.* ***(B)*** *Confocal maximum intensity projection images showing interactions of HUVECs and DPSCs. HUVECs were stained with UEA (green), actin was stained with phalloidin rhodamine (red), and nuclei with DAPI (blue). DPSCs joining the HUVEC network are pointed with white arrows. Scale bar is 200 µm.*

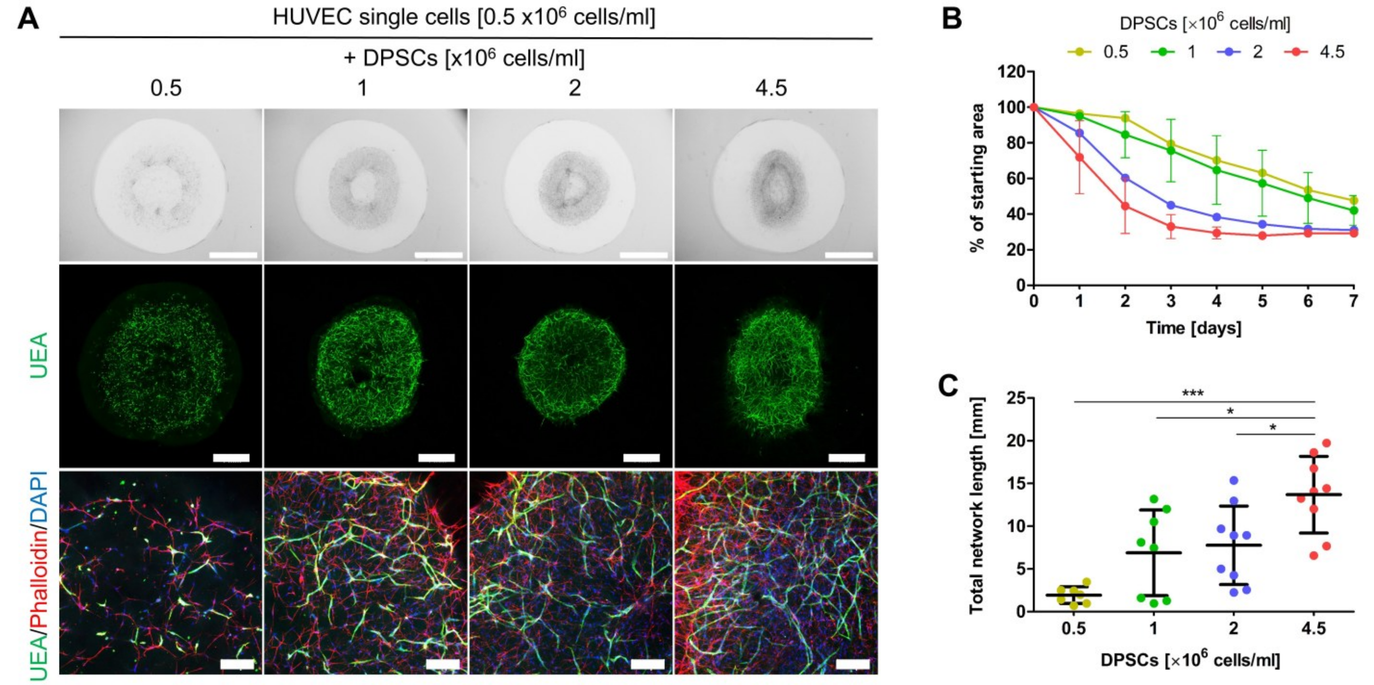

Figure S2: Vascular network formation in co-cultures of HUVECs and DPSCs in 3D Matrigel in EGM2. ***(A)*** *Bright field images of HUVECs (0.5 x 10^6 cells/ml) embedded in Matrigel with 0.5, 1, 2, or 4.5 x 10^6 DPSCs/ml (upper panel), maximum intensity projections of confocal z-stack images showing the whole hydrogel (middle panel), and maximum intensity projections of z-stack confocal images showing a detail (lower panel) after 7 days of culture. Samples were stained with Ulex Europaeus Agglutinin (UEA, green), phalloidin rhodamine (red), and DAPI (blue). Scale bars are 2 mm (upper panel), 1 mm (middle panel), and 200 µm (lower panel).* ***(B)*** *Changes of area covered with 3D Martigel during the course of 7 days when HUVECs were seeded with 0.5 x 10^6 (n = 1), 1 x 10^6 (n = 2), 2 x 10^6 (n = 1), and 4.5 x 10^6 DPSCs/ml (n = 2). Data are presented as percentage of starting area.* ***(C)*** *Total length of vascular network when HUVECs were seeded with 0.5 x 10^6 (n = 7), 1 x 10^6 (n = 8), 2 x 10^6 (n = 9), and 4.5 x 10^6 DPSCs/ml (n = 9). Data are shown as mean with SD. One-way ANOVA with Bonferroni's multiple comparison post hoc test was used for statistical analysis. * $p < 0.05$, *** $p < 0.001$.*

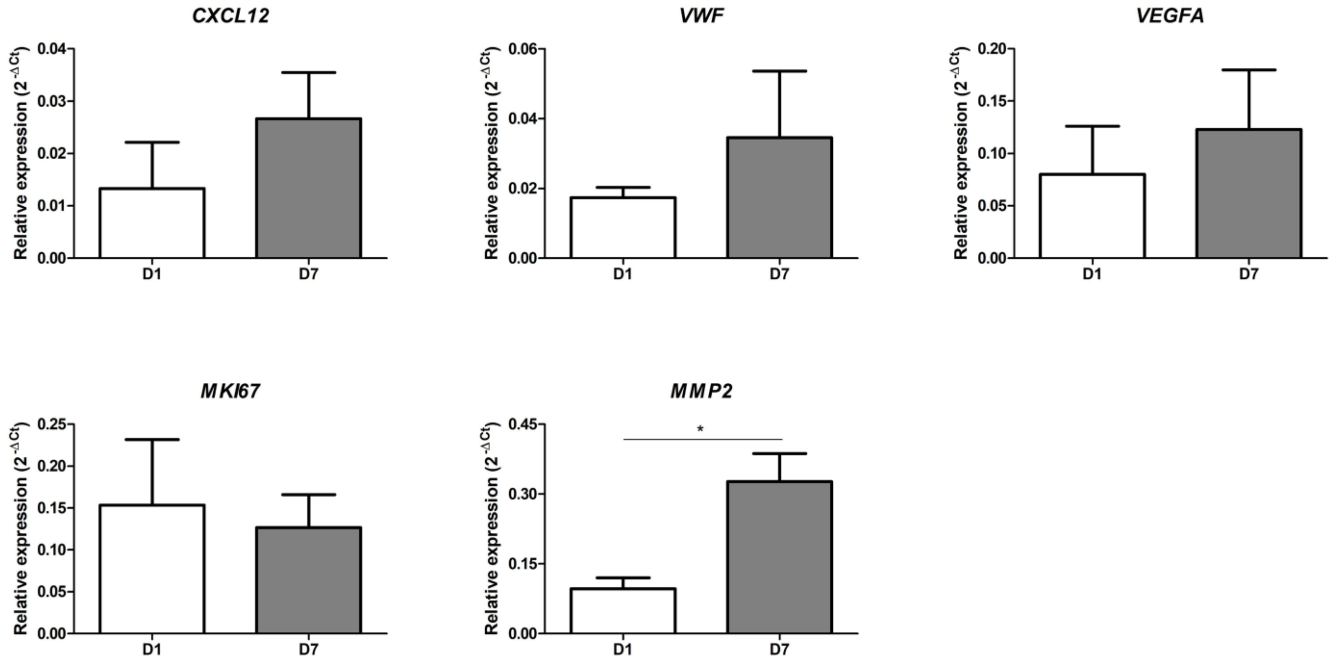

Figure S3: Real-time PCR analysis. *Relative mRNA expression of CXCL12, VWF, VEGFA, MKI67, and MMP2 in co-cultures of HUVEC spheroids and DPSCs in Matrigel in EGM2 at day 1 (D1) and day 7 (D7) of culture. Data are presented as mean + SEM. Unpaired t test was used for statistical analysis. * $p < 0.05$.*

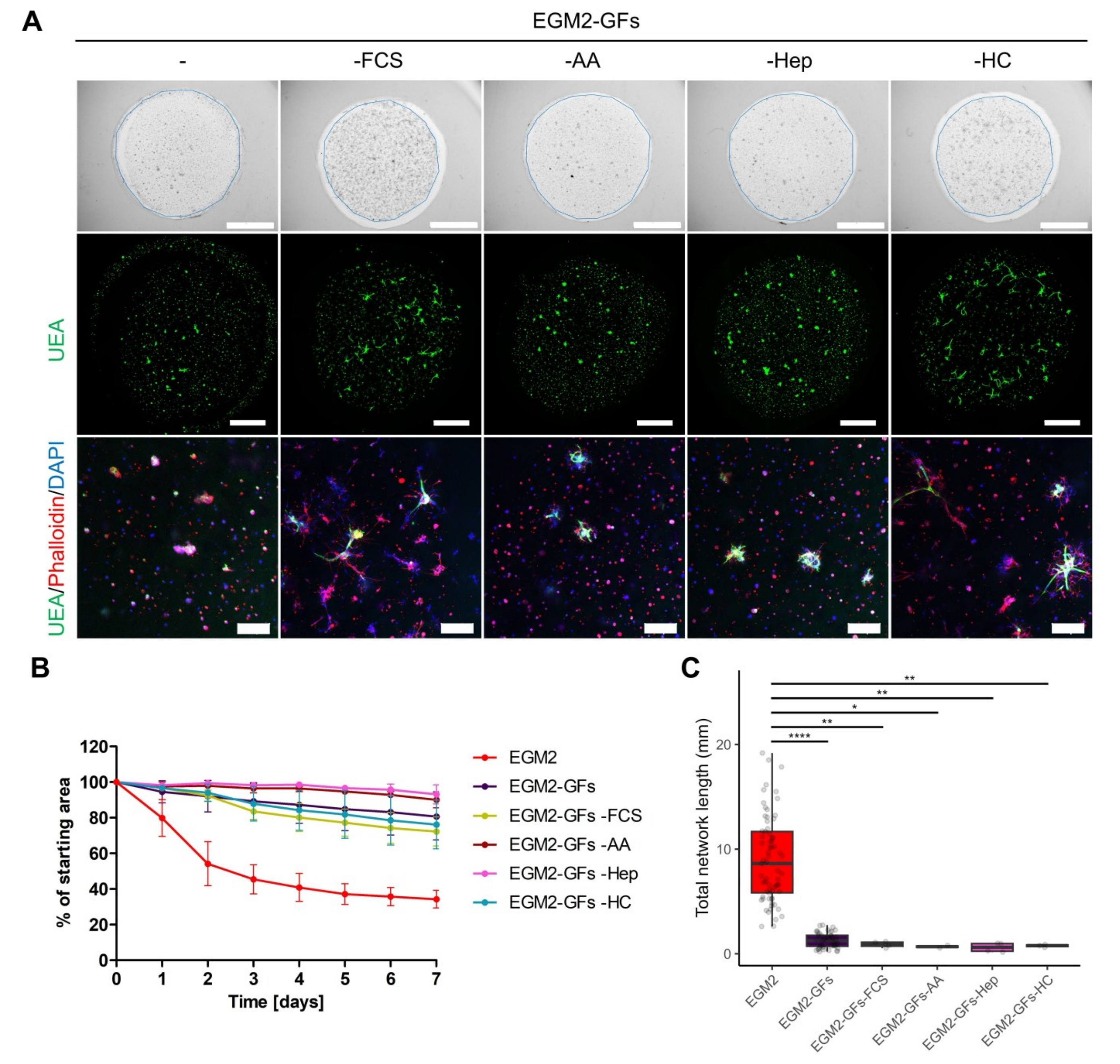

Figure S4: Effect of EGM2 supplements fetal calf serum (FCS), ascorbic acid (AA), heparin (Hep) and hydrocortisone (HC) on HUVEC network formation in co-cultures with DPSCs in 3D Matrigel. (A) *HUVECs in the form of spheroids with DPSCs at day 7 of cultivation in media: EGM2 without growth factors (EGM2-GFs), EGM2 without GFs and FCS (-FCS), EGM2 without GFs and AA (-AA), EGM2 without GFs and Hep (-Hep), EGM2 without GFs and HC (-HC). Bright field images of 3D Matrigel (upper), maximum intensity projections of tiled confocal z-stack images of 3D Matrigel (middle), maximum intensity projections of z-stack confocal images (lower). Staining for endothelial marker Ulex Europaeus Agglutinin I (UEA, green), actin stained with phalloidin rhodamine (red) and nuclei with DAPI (blue). Scale bar is 2 mm (upper), 1 mm (middle), 200 µm (lower).* ***(B)*** *Area covered with 3D Matrigel during the course of 7 days in EGM2 (n = 16), EGM2-GFs (n = 6), -FCS (n = 3), -AA (n = 3), -Hep (n = 3) and -HC (n = 4). Data are shown as mean ± SD.* ***(C)*** *Total length of formed HUVEC network in EGM2 (n = 69), EGM2-GFs (n = 48), EGM2-GFs-FCS (n = 7), EGM2-GFs-AA (n = 4), EGM2-GFs-Hep (n = 7) and -HC (n = 10). Data are shown as Tukey-style boxplots with the median, IQR, whiskers (1.5 × IQR) and outliers. Each of the tested media was compared to EGM2 and Mann Whitney test was used for statistical analysis.* $* p < 0.05$, $** p < 0.01$, $**** p < 0.0001$.

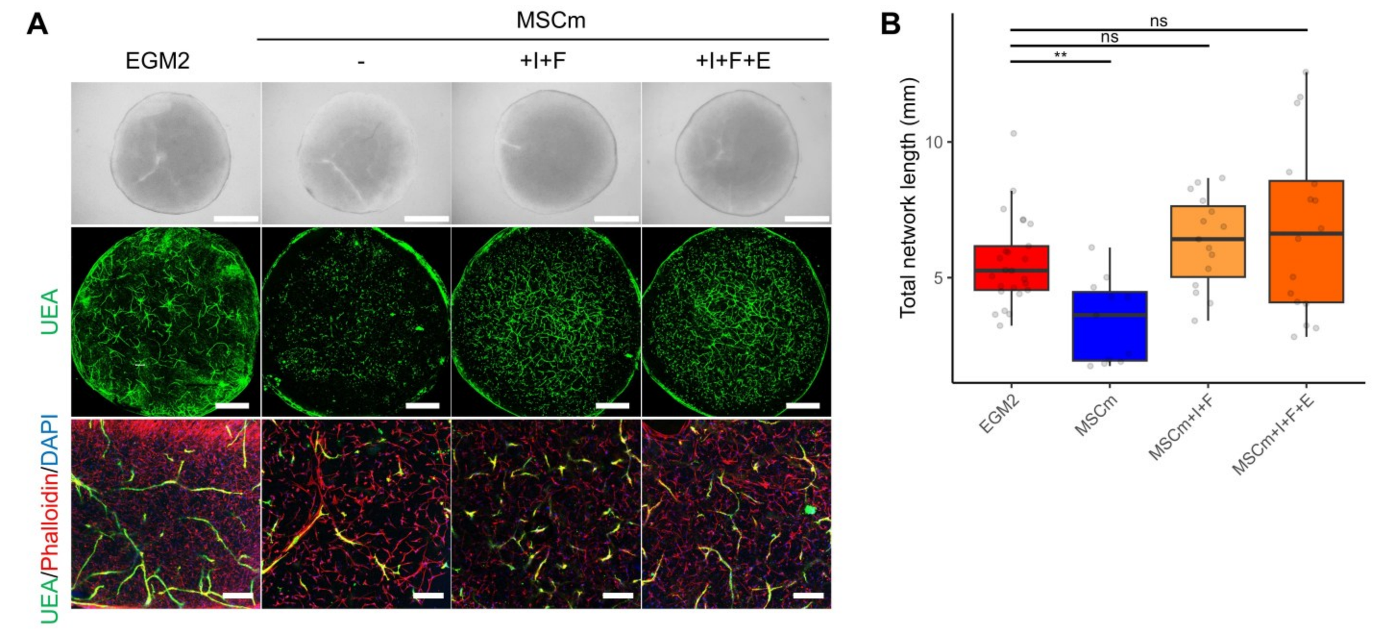

Figure S5: Addition of growth factors IGF1 and FGF2 to MSC medium can improve HUVEC network formation in co-cultures with DPSCs in GelMA. (A) *Bright field images (upper panel), confocal maximum intensity projection images showing the whole hydrogel (middle panel) and a detail (lower panel) of HUVEC spheroids in co-culture with DPSCs after 7 days of culture. Samples were stained with Ulex Europaeus Agglutinin (UEA, green), phalloidin rhodamine (red), and DAPI (blue). Scale bars are 2 mm (upper panel), 1 mm (middle panel), and 200 µm (lower panel).* ***(B)*** *Total length of HUVEC network in EGM2 (n = 25), MSC medium (n = 11), MSC medium supplemented with IGF1 and FGF2 (I+F, n = 17), and MSC medium supplemented with IGF1, FGF2, and EGF (I+F+E, n = 19). Data are shown as Tukey-style boxplots with the median, IQR, whiskers (1.5 × IQR) and outliers. Each of the tested media was compared to EGM2 and Mann Whitney test was used for statistical analysis. ** $p < 0.01$.*

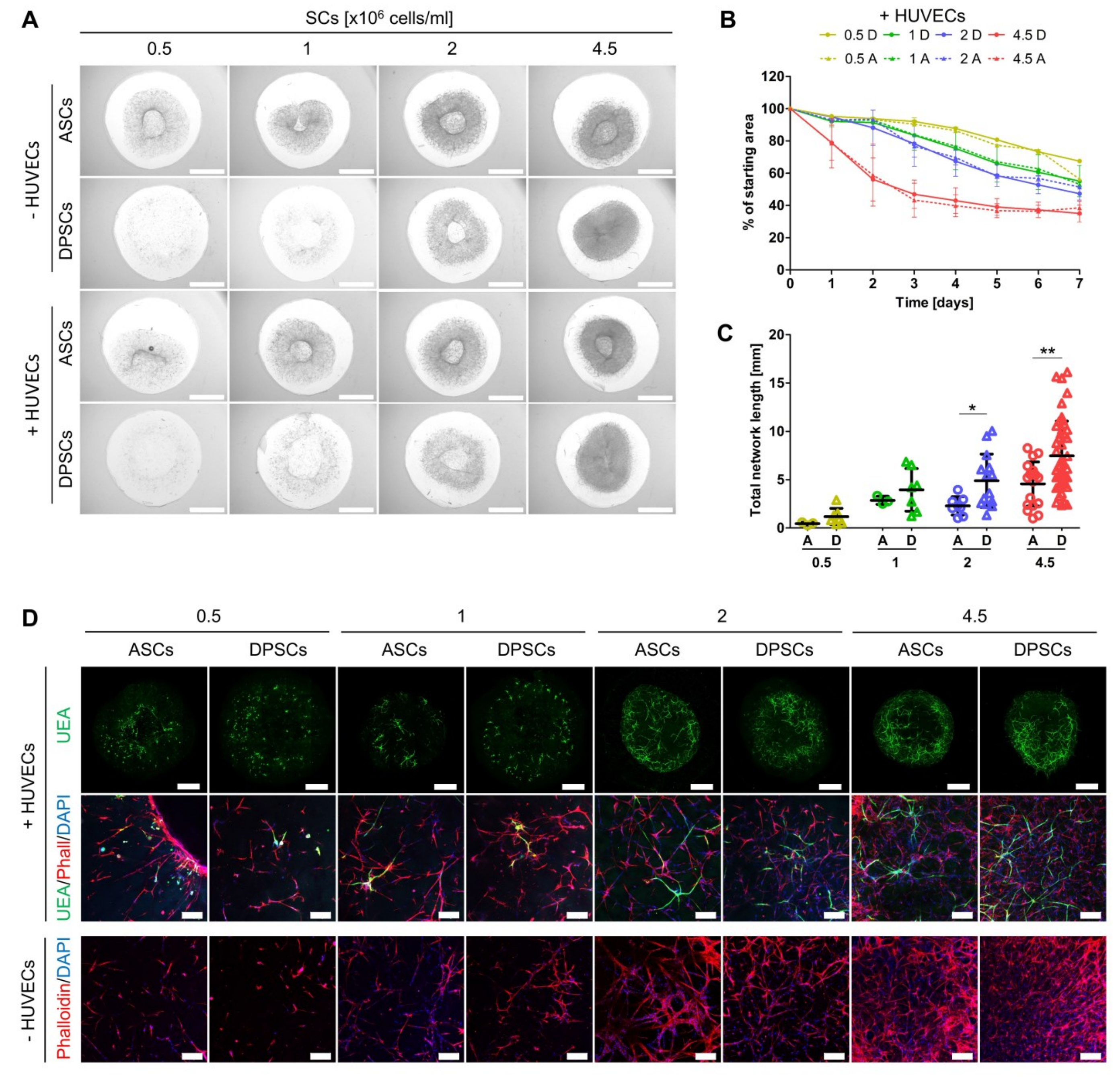

Figure S6: Different concentrations of ASCs and DPSCs embedded without or with HUVEC spheroids in 3D Matrigel. ***(A)*** *Bright field images showing Matrigel with stromal cells (SCs; ASCs or DPSCs) embedded without or with HUVEC spheroids after 7 days of cultivation. Stromal cells were seeded at the concentration 0.5, 1, 2 or 4.5 M/ml.* ***(B)*** *Area covered with 3D Matrigel during the course of 7 days shown as percentage of starting area. DPSCs - full line, ASCs - dashed line. Data are shown as mean ± SD.* ***(C)*** *Total HUVEC network length formed in co-culture with different concentrations of ASCs or DPSCs. Data are shown as mean ± SD. Unpaired t test was used for statistical analysis. * $p < 0.05$, ** $p < 0.01$.* ***(D)*** *Maximum intensity projections of confocal z-stack images showing ASCs or DPSCs at concentrations 0.5, 1, 2, and 4.5 x 10^6 cells/ml with or without HUVEC spheroids within Matrigel after 7 days of culture. Staining was done using the endothelial marker Ulex Europaeus Agglutinin I (UEA, green), actin was stained with phalloidin rhodamine (red), and nuclei with DAPI (blue). Scale bars are 1 mm (top), 200 µm (middle and bottom).*

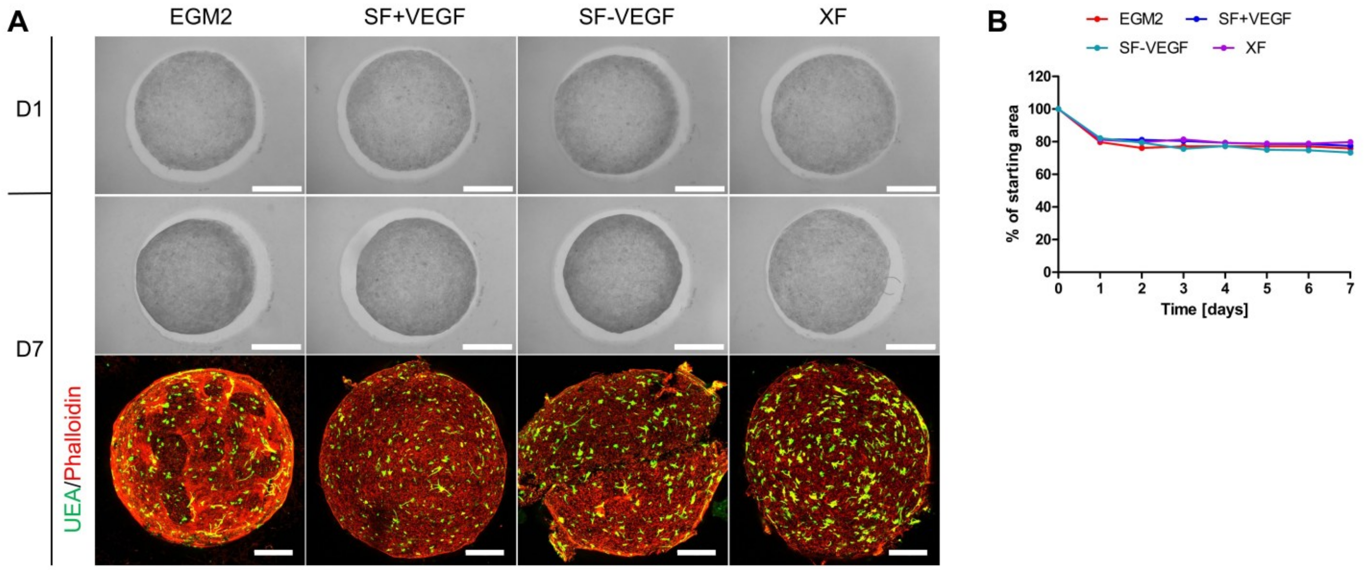

Figure S7*: *HUVEC vascular network formation in co-cultures with DPSCs in chemically defined media in diluted VitroGel Angiogenesis Assay HC Kit (AKK1). ***(A)*** *Bright field images of HUVECs embedded together with DPSCs in 3D AAK1 diluted 1:1 with Dilution Solution at day 1 (D1) and day 7 (D7) of culture in EGM2 medium (control), serum-free (SF) medium with VEGF (SF+VEGF) or without VEGF (SF-VEGF), and xeno-free (XF) medium. Maximum intensity projections of z-stack confocal images showing HUVEC network stained with Ulex Europaeus Agglutinin (UEA, green) and actin stained with Phalloidin Rhodamine (red). Scale bar is 2 mm on the bright field images and 1 mm on the maximum intensity projection images.* ***(B)*** *Changes of area covered with hydrogel during the course of 7 days shown as percentage of starting area. N = 1.*

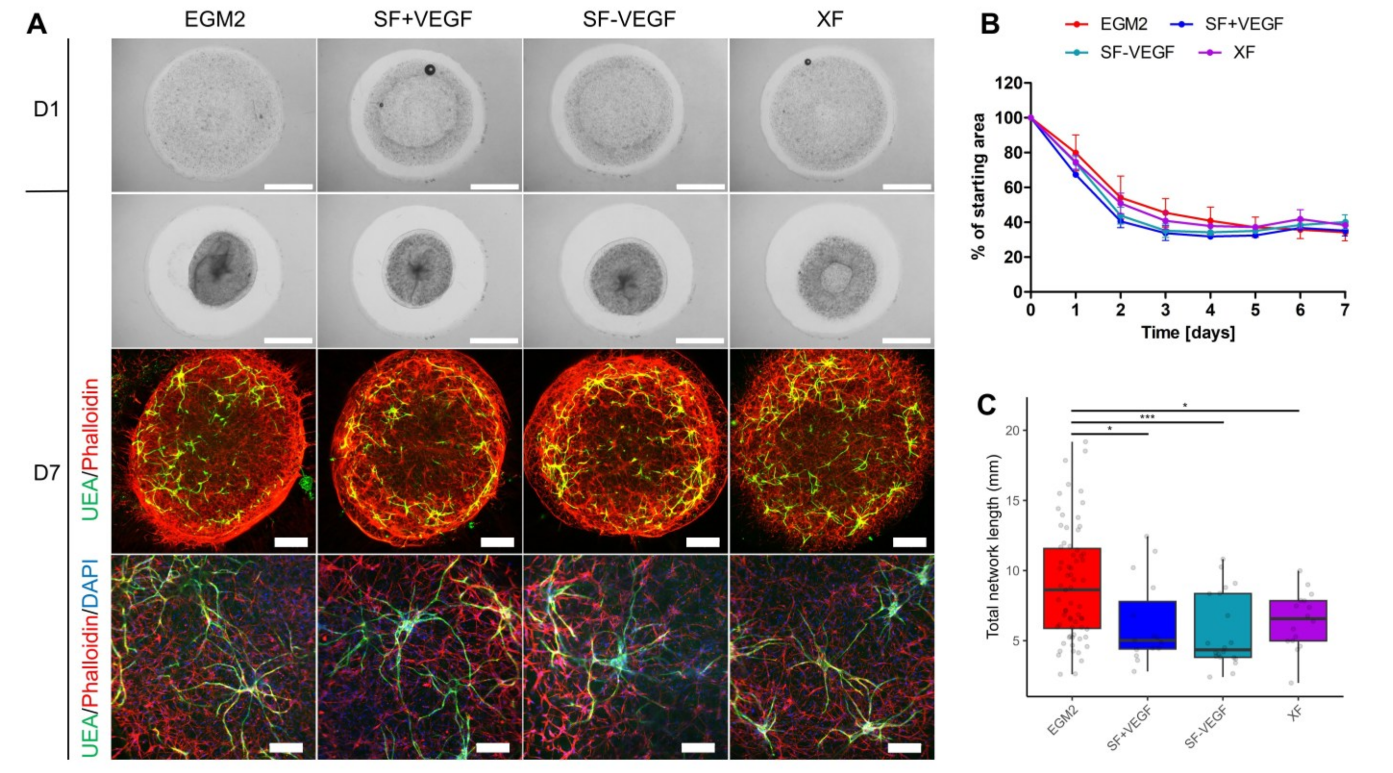

***Figure S8**: **HUVEC vascular network formation in co-culture with DPSCs within Matrigel in chemically defined media.** **(A)** Bright field images of HUVEC spheroids (0.5 x 10^6 cells/ml) embedded with 4.5 x 10^6 DPSCs/ml at day 1 (D1) of culture and bright field images, maximum intensity projections of confocal z-stack images showing the whole hydrogel, and maximum intensity projections of z-stack confocal images showing a detail at day 7 (D7) of culture. Samples were stained with Ulex Europaeus Agglutinin (UEA, green), phalloidin rhodamine (red), and DAPI (blue). Scale bars are 2 mm on the bright field images, 500 µm on the confocal images of the whole hydrogels, and 200 µm on the images showing a detail. Used media are EGM2 (control), EGM2 with FCS substituted with B27 (serum free with VEGF, SF+VEGF), EGM2 with FCS substituted with B27 and without VEGF (serum-free without VEGF, SF-VEGF), and EGM2 medium without FCS and VEGF (xeno-free, XF). **(B)** Change of area covered with Matrigel during the course of 7 days shown as a percentage of the starting area. EGM2 (n = 16), SF+VEGF (n = 2), SF-VEGF (n = 2), and XF (n= 2). Data are shown as mean ± SD. **(C)** Total length of HUVEC network in the media EGM2 (n = 67), SF+VEGF (n = 15), SF-VEGF (n = 20), and XF (n= 16)). Data are shown as Tukey-style boxplots with the median, IQR, whiskers (1.5 × IQR) and outliers. Each of the tested media was compared to EGM2 and Mann Whitney test was used for statistical analysis. * $p < 0.05$, *** $p < 0.001$.*

Table S1: EGM2-based media composition. 20 ng/ml IGF1, 10 ng/ml FGF2, 5 ng/ml EGF, 22.5 µg/ml heparin (Hep), 1 µg/ml ascorbic acid (AA), 0.2 µg/ml hydrocortisone (HC), 1 % Penicillin-Streptomycin (P/S).

**Acronym**	**Basal medium**	**Serum**	**Growth factors**	**Supplements**	**Figure**
EGM2	EBM	2 % FCS	IGF, FGF2, EGF, 0.5 ng/ml VEGF	Hep, AA, HC, P/S	1, 2, 3, 4, 5, 6, 7, S1, S2, S3, S4, S5, S6, S7, S8
EGM2-GFs	EBM	2 % FCS	-	Hep, AA, HC, P/S	3, 4, S4
Hep+AA+HC (-FCS)	EBM	-	-	Hep, AA, HC, P/S	S4
FCS+Hep+HC (-AA)	EBM	2 % FCS	-	Hep, HC, P/S	S4
FCS+AA+HC (-Hep)	EBM	2 % FCS	-	AA, HC, P/S	S4
FCS+Hep+AA (-HC)	EBM	2 % FCS	-	Hep, AA, P/S	S4
VEGF_0.5	EBM	2 % FCS	0.5 ng/ml VEGF	Hep, AA, HC, P/S	3
VEGF_10	EBM	2 % FCS	10 ng/ml VEGF	Hep, AA, HC, P/S	3
VEGF_50	EBM	2 % FCS	50 ng/ml VEGF	Hep, AA, HC, P/S	3
VEGF_100	EBM	2 % FCS	100 ng/ml VEGF	Hep, AA, HC, P/S	3
IGF1	EBM	2 % FCS	IGF1	Hep, AA, HC, P/S	4
FGF2	EBM	2 % FCS	FGF2	Hep, AA, HC, P/S	4
EGF	EBM	2 % FCS	EGF	Hep, AA, HC, P/S	4
I+F	EBM	2 % FCS	IGF1, FGF2	Hep, AA, HC, P/S	4
I+F+E	EBM	2 % FCS	IGF1, FGF2, EGF	Hep, AA, HC, P/S	4
I+F+V	EBM	2 % FCS	IGF1, FGF2, 0.5 ng/ml VEGF	Hep, AA, HC, P/S	4

Table S2: MSC medium-based media composition. 20 ng/ml IGF1, 10 ng/ml FGF2, 5 ng/ml EGF, 1× GlutaMAX-1, 1 % Penicillin-Streptomycin (P/S).

Acronym	Basal medium	Serum	Growth factors	Supplements	Figure
MSCm	DMEM/F-12	10 % FBS	-	GlutaMAX, P/S	2, 5, 6, S5
IGF1	DMEM/F-12	10 % FBS	IGF1	GlutaMAX, P/S	5, 6
FGF2	DMEM/F-12	10 % FBS	FGF2	GlutaMAX, P/S	5, 6
EGF	DMEM/F-12	10 % FBS	EGF	GlutaMAX, P/S	5, 6
I+F	DMEM/F-12	10 % FBS	IGF1, FGF2	GlutaMAX, P/S	5, 6, S5
I+F+E	DMEM/F-12	10 % FBS	IGF1, FGF2, EGF	GlutaMAX, P/S	5, 6, S5

Table S3: Chemically defined and xeno-free media. B27 (B-27 Supplement (50X), minus vitamin A, Gibco, 12587010), 20 ng/ml IGF1, 10 ng/ml FGF2, 5 ng/ml EGF, 0.5 ng/ml VEGF, 22.5 µg/ml heparin (Hep), 1 µg/ml ascorbic acid (AA), 0.2 µg/ml hydrocortisone (HC), 1 % Penicillin-Streptomycin (P/S). Serum-free (SF), xeno-free (XF).

Acronym	Basal medium	B27	Growth factors	Supplements	Figure
SF+VEGF	EBM	1×	IGF, FGF2, EGF, VEGF	Hep, AA, HC, P/S	7, S7, S8
SF-VEGF	EBM	1×	IGF, FGF2, EGF	Hep, AA, HC, P/S	7, S7, S8
XF	EBM	-	IGF, FGF2, EGF	Hep, AA, HC, P/S	7, S7, S8

Table S4: The sequences of primers used for qPCR.

Gene	Primer sequence
CXCL12	Forward: 5'-ATTCTCAACACTCCAAACTGTGC-3'<br>Reverse: 5'-ACTTTAGCTTCGGGTCAATGC-3'
VWF	Forward: 5'-CCTTGACCTCGGACCCTTATG-3'<br>Reverse: 5'-GATGCCCGTTCACACCACT-3'
VEGFA	Forward: 5'-CGAGTACATCTTCAAGCCATCC-3'<br>Reverse: 5'-TGGTGAGGTTTGATCCGC-3'
MKI67	Forward: 5'-AGCGTGCAGGAATCTAATGC-3'<br>Reverse: 5'-CTACTGATGGTTTAGGCGTGTG-3'
MMP2	Forward: 5'-GATACCCCTTTGACGGTAAGGA-3'<br>Reverse: 5'-CCTTCTCCCAAGGTCCATAGC-3'
GAPDH	Forward: 5'-AGGTGAAGGTCGGAGTCAAC-3'<br>Reverse: 5'-CCATGTAGTTGAGGTCAATGAAG-3'
